# Comparative analysis of chloroplast genome and new insights into phylogenetic relationships of *Ajuga* and common adulterants

**DOI:** 10.3389/fpls.2023.1251829

**Published:** 2023-10-25

**Authors:** Mingyue Shang, Jiale Wang, Guona Dai, Jiamei Zheng, Binbin Liao, Jing Wang, Baozhong Duan

**Affiliations:** ^1^ College of Pharmaceutical Science, Dali University, Dali, China; ^2^ College of Life Science, Northeast Forestry University, Harbin, China

**Keywords:** chloroplast genome, *ajuga bracteosa*, molecular marker, species identification, phylogenetic

## Abstract

**Introduction:**

The potential contamination of herbal medicinal products poses a significant concern for consumer health. Given the limited availability of genetic information concerning Ajuga species, it becomes imperative to incorporate supplementary molecular markers to enhance and ensure accurate species identification.

**Methods:**

In this study, the chloroplast (cp) genomes of seven species of the genus Ajuag were sequenced, de novo assembled and characterized.

**Results:**

exhibiting lengths ranging from 150,342 bp to 150,472 bp, encompassing 86 - 88 protein-coding genes (PCGs), 35 - 37 transfer RNA, and eight ribosomal RNA. The repetitive sequences, codon uses, and cp genomes of seven species were highly conserved, and PCGs were the reliable molecular markers for investigating the phylogenetic relationship within the Ajuga genus. Moreover, four mutation hotspot regions (accD-psaI, atpH-atpI, ndhC-trnV(UAC), and ndhF-rpl23) were identified within cp genomes of Ajuga, which could help distinguish A. bracteosa and its contaminants. Based on cp genomes and PCGs, the phylogenetic tree preliminary confirmed the position of Ajuga within the Lamiaceae family. It strongly supported a sister relationship between Subsect. Genevense and Subsect. Biflorae, suggesting the merger of Subsect. Biflorae and Subsect. Genevenses into one group rather than maintaining separate categorizations. Additionally, molecular clock analysis estimated the divergence time of Ajuga to be around 7.78 million years ago.

**Discussion:**

The species authentication, phylogeny, and evolution analyses of the Ajuga species may benefit from the above findings.

## 1 Introduction

The *Ajuga* genus is a member of the Lamiaceae family and encompasses numerous economically and medically significant plants. Many *Ajuga* species have been employed in Traditional Chinese Medicine for relieving cough, reducing sputum, and arresting bleeding (Dai et al., 2010). Among the *Ajuga* species, *A. bracteosa* is particularly prevalent and extensively utilized in folk medicine (Millen et al., 2001). Previous investigation has revealed that the morphologies of most *Ajuga* species are similar and indistinguishable, potentially leading to misclassification (Talebi et al., 2019). According to the World Health Organization, adulterating herbal products threatens consumer safety (Duan et al., 2017). In recent years, molecular identification techniques, particularly molecular markers, have made notable advancements in traditional Chinese medicine (TCM), which were used to recognize variations among individuals of distinct species or populations (Cheng et al., 2018; Liu et al., 2019). Previous studies have employed the nuclear ribosomal DNA internal transcribed spacer (ITS) 2 to identify certain *Ajuga* species and related taxa, such as *A. ciliate*, *A. decumbens*, and *A. lupulina* (Han et al., 2008). Nonetheless, some frequent adulterants were not probed, and single-locus DNA barcodes inherently possess limitations.

Additionally, elucidating the taxonomic relationships between species of the genus *Ajuga* is crucial for understanding and harnessing the medicinal properties of the different species. However, the presence of similar morphological features coupled with the dearth of molecular information has prevented the accurate identification of taxonomic relationships in this genus. Hence, it is essential to develop a more accurate and effective method for identifying and classifying the *Ajuga* taxa.

The organelle chloroplast (cp) is vital in plant photosynthesis and biochemical processes (Zoschke and Bock, 2018). Compared to conventional DNA portions, the cp genome exhibits higher conservation with slight variations, making it applicable in various research areas, including species authentification and developing DNA markers (Qiao et al., 2016). Plastid genome analysis has been widely employed to identify *Paris, Isodon*, and respective adulterants and clarify phylogenetic relationships (Jiang et al., 2022; Zhou et al., 2022). Although previous studies have reported plastid genomes of *Ajuga* (Tao et al., 2019), their focus was primarily on characterizing a single genome, and cp genomes have not been used to differentiate *Ajuga* taxa and their frequent adulterants. In addition, cp genomes may lead to a wrong inference of phylogenetic relationships due to DNA length disparity, gaps representing insertions/deletions (indels), and improper models of DNA evolution in merged datasets. Previous investigations have indicated that protein-coding genes (PCGs) offer an improved resolution for understanding phylogenetic relationships due to the genetic divergence in gene-encoding regions occurring at a slower rate compared to the non-coding areas (Lockhart and Penny, 2005; Cui et al., 2019). However, no reports exist on using PCGs to comprehend the interspecific relationships among the *Ajuga* species.

Herein, cp genomes of seven *Ajuga* species were sequenced, *de novo* assembled, and annotated. Subsequently, we compared the architecture and evolutionary connections of these genomes. This research aims (i) to contribute novel and full-length plastid genomes of *Ajuga* to understand more about the genome structure of relevant species, (ii) to expound the phylogenetic relationship of *Ajuga* by comparing genome sequences, and (iii) to develop promising DNA markers for distinguishing *A. bracteosa* from its contaminants. Our results significantly increase the genome information of *Ajuga*, facilitate evolutionary scrutiny and authentification of *Ajuga*, and ensure the safe utilization of *A. bracteosa.*

## 2 Materials and methods

### 2.1 Plant materials and DNA extraction

Fresh leaves from seven species, namely *A. forrestii, A. nubigena, A. campylantha, A. macrosperma, A. bracteosa, A. nipponensis*, and *A. ovalifolia*, were gathered from the Germplasm Resource Garden of Kunming Zhifen Biotechnology Co., Ltd, Yunnan, China (102°48′58″E, 24°49′55″N). The voucher specimens were identified by Professor Baozhong Duan and preserved at the herbarium of Dali University. Information on each sample is detailed in **Table S1** and **Figure S1**. Approximately one gram of fresh leaves of each species was collected, instantly frozen in liquid nitrogen, and stored for subsequent DNA extraction. Genomic DNA was extracted from samples with a Plant Genomic DNA kit (Tiangen, Beijing, China) following the manufacturer’s instructions. The extracted DNA was checked with high-sensitivity Qubit 4.0 fluorometry (Life Technologies, Inc.).

### 2.2 Genome sequencing, assembly, and annotation

To prepare sequencing libraries, a high-quality DNA sample of at least 30 microliters per individual was utilized, with a minimum concentration of 100 ng/μL. Illumina NovaSeq system (Illumina, San Diego, CA) was adopted to sequence libraries. 1 μg of purified DNA was fragmented and used to build PE libraries (insert size 250 bp). The paired-end sequencing reads were filtered to trim adapter sequences and low-quality bases using Toolkit_v2.3.3 software. The cp genome was assembled using GetOrganelle v.1.6.4, exploiting Blast v.2.5.0, SPAdes v.3.13.0, and Bowtie2 v.2.4.4 as dependencies (get_organelle_from_reads.py -1 R1.fq -2 R2.fq - o cp_output -R 15 -k 21,45,65,85,105 -F embplant) (Jin et al., 2020). All clean reads were mapped to the database, and then the mapping data were extracted based on similarity and coverage. Subsequently, the assembled contigs were visualized, and removed redundant sequences by Bandage v.0.8 to generate the complete circular cp genome (Wang J. et al., 2022). Finally, the reads were remapped to assemble the cp genome by Bowtie2, and Jellyfish v.2.2.3 was then used to determine the reverse repeat region boundary. Following assembly, CpGAVAS2 (http://47.96.249.172:16019/analyzer/annotate) (An et al., 2020) and GeSeq (https://chlorobox.mpimp-golm.mpg.de/geseq.html) (Liu et al., 2020) were employed to annotate the circular plastid genomes, which can be retrieved from the National Center for Biotechnology Information (NCBI) GenBank with accession numbers OR038698 to OR038704 (**Table 1**). IRscope toll (https://irscope.shinyapps.io/Chloroplot/) (Amiryousefi et al., 2018) was used to visualize gene maps.

**Table 1:** Summary of cp genome features.

Genome features	*A. forrestii*	*A. nubigena*	*A. campylantha*	*A. macrosperma*	*A. bracteosa*	*A. nipponensis*	*A. ovalifolia*
Total length (bp)	150472	150462	150453	150362	150342	150442	150443
LSC length (bp)	82170	82158	82152	82085	82080	82121	82160
SSC length (bp)	17160	17158	17171	17153	17174	17183	17179
IR length (bp)	25571	25573	25565	25562	25544	25569	25552
AT content (%)	61.7	61.7	61.7	61.7	61.7	61.7	61.7
Total GC content (%)	38.3	38.3	38.3	38.3	38.3	38.3	38.3
GC content in LSC (%)	36.4	36.4	36.4	36.4	36.4	36.4	36.4
GC content in SSC (%)	32.1	32.3	32.2	32.2	32.2	32.2	32.3
GC content in IR (%)	43.3	43.3	43.3	43.3	43.3	43.3	43.3
Gene number	129	130	129	131	131	133	133
tRNA gene number	35	36	35	37	37	37	37
rRNA gene number	8	8	8	8	8	8	8
Protein-coding gene number	86	86	86	86	86	88	88
GenBank accession	OR038698	OR038699	OR038700	OR038701	OR038702	OR038703	OR038704

### 2.3 Repeat analysis and comparative analyses

Four types of dispersed repeat sequences, i.e., Forward (F), Reverse (R), Palindromic (P), and Complementary (C), were identified with the REPuter tool (https://bibiserv.cebitec.uni-bielefeld.de/reputer/) with a minimal repeat of 30 bp and a similarity threshold of 90% between repeat pairs (Kurtz et al., 2001). In addition, simple sequence repeats (SSRs) were analyzed with MISA software (http://pgrc.ipk-gatersleben.de/misa/) (Beier et al., 2017), with thresholds of ‘10’ in mono-, ‘5’ in di-, ‘4’ in tri-, and ‘3’ in tetra-, penta-, and hexa- nucleotide motifs. Geneious 9.0.2 software was used to analyze GC content, genome size, tRNA, and repeat content (Kearse et al., 2012), while CodonW v.1.4.2 was utilized to assess codon usage bias through six values, including effective number of codons (Enc), GC content of synonymous third codon positions (GC3s), codon adaptation index (CAI), frequency of optimal codons (Fop), relative synonymous codon usage (RSCU) and codon bias index (CBI). The RSCU values were visualized through a heatmap generated with Tbtools (Chen et al., 2020). The IRscope tool (https://irscope.shinyapps.io/irapp/) was employed to investigate the contraction and expansion of inverted repeat (IR) regions at the junctions of plastid genomes (Amiryousefi et al., 2018). The genome sequences were also analyzed based on the annotation information of *A. bracteosa* (GenBank NC068635.1) using the mVISTA program in Shuffle LAGAN mode (Poliakov et al., 2014). Nucleotide variability (Pi) across cp genome sequences was calculated with DnaSP v.6.12.03, and specific settings were: 200 bp of step size and 600 bp of window length (Wang J. et al., 2022). Pi exceeding 0.008 was considered a mutation hotspot.

### 2.4 Identification and validation of barcode for species discrimination

The intergenic spacers (IGS) were obtained from seven *Ajuga* species with PhyloSuite v1.2.2 (Zhang et al., 2019). Primers were designed based on the variable intergenic regions using Snapgene 6.2.1 (Snapgene, Insightful Science, available at http://www.snapgene.com, last used in 2023). PCR amplifications were conducted in a final volume of 25 μL, consisting of 12.5 μL of 2×Taq Plus PCR Master Mix, 1 μL of each primer, 2 μL of template DNA, and 8.5 μL of ddH_2_O (Mei5 Biotechnology, Co., Ltd). All amplifications were performed using a RePure-A PCR system (Applied Biogener, Hangzhou, China) under the following conditions: an initial denaturation at 94°C for 3 min, followed by 35 cycles of 94°C for 30 s, 55°C for 30 s, and 72°C for 1 min, with a final extension at 72°C for 5 min. PCR products were examined by 1% agarose gel electrophoresis to confirm the amplification of the target fragments. The purified PCR products were sequenced in both directions on a 3730XL DNA Sequencer (Applied Biosystems, Waltham, USA) using the same primers at Sangon Biotech Co., Ltd. (Shanghai, China).

### 2.5 Phylogeny and divergence time estimation

The phylogenetic analysis involved a total of 35 taxa, consisting of 28 species obtained from NCBI (refer to **Table S2**) and seven species that we newly sequenced, as detailed in **Table 1**. The selection of these species for phylogenetic analysis is based on the classification system of Lamiaceae within the Angiosperm Phylogeny Group IV system (APG IV). Additionally, two species, *Callicarpa macrophylla* (GenBank NC058323.1) and *C. arborea* (GenBank NC058321.1) served as outgroups. The 68 common PCGs of 35 cp genomes were extracted based on the annotation files. The aligned sequences were generated using the MAFFT program and verified manually. Phylogenetic analysis was performed using Maximum likelihood (ML), Bayesian inference (BI), and Neighbor joining (NJ). For the ML tree reconstruction, IQtree was employed with default settings, 1,000 iterations, and 1,000 replications. Model selection was based on the best-fit approach (Katoh and Standley, 2013). BI analysis was performed using MrBayes v.3.2.6 (Ronquist et al., 2012). The most appropriate model for sequence substitution in plastid genomes (GTR + G + I) and PCGs (GTR + G) was determined using MEGA X v.10.2.6 (Mao et al., 2023). The parameters were set for five million generations, with sampling every 1,000 generations. The initial 25% of each run was discarded as burn-in (Jiang et al., 2023). Moreover, the NJ tree was inferred with MEGA X v.10.2.6 and subjected to the bootstrap test of 1,000 repetitions (Kumar et al., 2018).

To estimate divergence time, a molecular clock tree was constructed based on an ML tree with MEGA X. The corresponding divergence times were determined using the TimeTree Resource (RRID: SCR_021162) (Kumar et al., 2017), and seven calibration points were utilized to calculated the divergence times for each node as follows: (F1) 33.3 - 72.4 million years ago (Mya) for the root node, (F2) 19.4 - 39.9 Mya for Ajugoideae + Lamioideae, (F3) 14.0 - 42.8 Mya for Lamioideae stem age, (F4) 1.2 - 62.7 Mya for Nepetoideae stem age, (F5) 12.4 - 47.2 Mya for *Salvia prionitis* + *Prunella vulgaris*, (F6) 0.6 - 19.2 Mya for *Leonurus sibiricus* + *L. amplexicaule*, and (F7) 5.2 - 9.6 Mya for *Pogostemon cablin* + *P. septentrionalis.*

## 3 Results and discussion

### 3.1 Genome structure

#### 3.1.1 Genome characteristic

**Figure 1:**
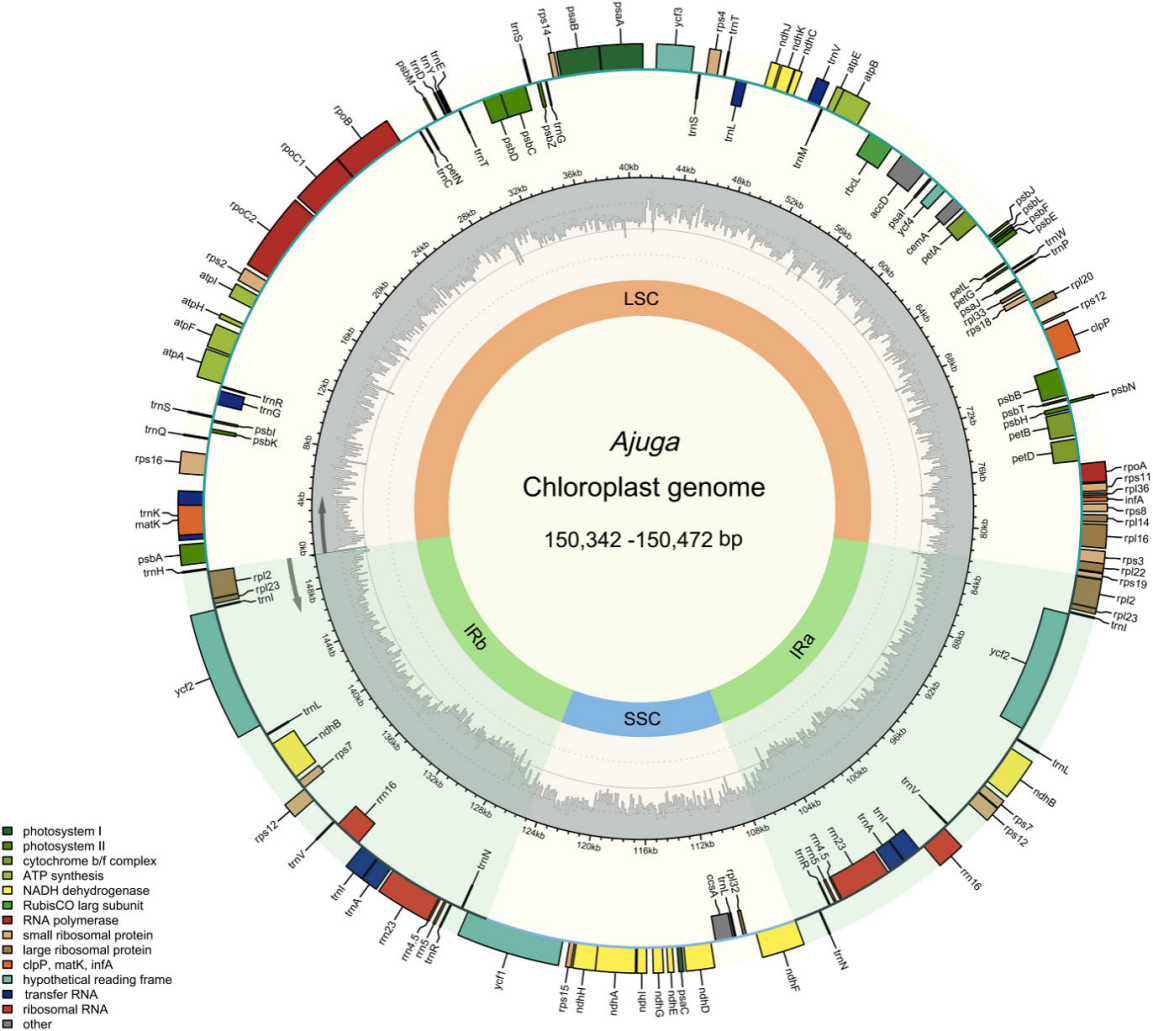
The cp genome map of *Ajuga*.

Around 2.51 - 4.54 Gb data were obtained from each species. The *A. macrosperma* and *A. ovalifolia* were reported for the first time. As shown in **Figure 1**, cp genomes of seven species are circular DNAs ranging from 150,342 bp to 150,472 bp and exhibit the typical quadripartite structure commonly observed in most angiosperm cp (Palmer, 1985; Henriquez et al., 2020; Yun and Kim, 2022), consisted of two IRs (IRa and IRb) separated by large single copy (LSC) and small single copy (SSC) regions, respectively. The length of LSC regions ranged from 82,080 bp (*A. campylantha*) to 82,170 bp (*A. ovalifolia*), SSC regions ranged from 17,183 bp (*A. nubigena*) to 17,153 bp (*A. macrosperma*), and IRa and IRb regions ranged from 25,573 bp (*A. nipponensis*) to 25,544 bp (*A. campylantha*). The overall GC content of seven *Ajuga* cp genomes was 38.3%, largely concordant with the prior study of Tao et al. (Tao et al., 2019), indicating a high degree of conservation among *Ajuga* species’ cp genomes. Notably, the IR regions exhibited a significantly higher GC content of 43.3% in contrast to the LSC (36.4%) and SSC (32.1% - 32.3%) regions. This discordance may be attributed to the fact that four ribosomal RNA (rRNA) genes (*rrn23, rrn16, rrn5, rrn4.5*) with high GC content were located in the IR regions, which was similar to the most plant species (Yan et al., 2019; Gui et al., 2020; Chen et al., 2022).

In addition, 129 - 133 genes were detected, including 86 - 88 PCGs, 35 - 37 transfer RNAs (tRNAs), and eight ribosomal RNAs (**Table 1**). Variations in gene numbers across these species can be attributed to the expansion and contraction of IRs. These genes can be categorized into three groups: 45 associated with photosynthesis, 27 involved in self-replication, and the remaining genes serving various other functions. Notably, three genes (*chIB*, *chIL*, *ycf68*) were absent in the seven *Ajuga* species. The missing *chIB* and *chIL* may be a distinctive feature of flowering plants (Jansen et al., 2007). Furthermore, the *ycf68* gene was also absent in the cp genomes of *Miscanthus sinensis* and *M. floridulus* (Sheng et al., 2021). In addition to these observations, it’s worth mentioning that two tRNAs, *trnF-GAA* and *trnfM-CAU*, were absent in three *Ajuga* species. Specifically, *A. nubigena* lacked the *trnF-GAA* gene, while *trnF-GAA* and *trnfM-CAU* genes were missing in *A. forrestii* and *A. campylantha.* These findings revealed that the structure of cp genomes was highly conserved in *Ajuga* species, albeit with some alterations that have accrued during the angiosperm evolution, which was also supported by previous studies (Millen et al., 2001).

Furthermore, the seven *Ajuga* species exhibit a similar number and types of introns. As illustrated in **Table S3**, each of 18 genes had a single intron, *trnI-GAU* (×2), *trnA-UGC* (×2), *rpl2* (×2), and *ndhB* (×2) were in IR, and genes *trnK-UUU*, *trnG-UCC*, *trnL-UAA*, *trnV-UAA*, *rpl16*, *rps16*, *petB, rpoC1*, *petD*, and *atpF* were in LSC region, while *ndhA* was only present in SSC. Besides, *clpP* and *ycf3* contain double introns, consistent with previous findings (Gong et al., 2022). It is unsurprising given that most angiosperm plastid genomes exhibit highly conserved structure and gene composition at the genus level (Wicke et al., 2011; Chen et al., 2021; Namgung et al., 2021).

#### 3.1.2 Inverted repeats regions contraction and expansion

**Figure 2:**
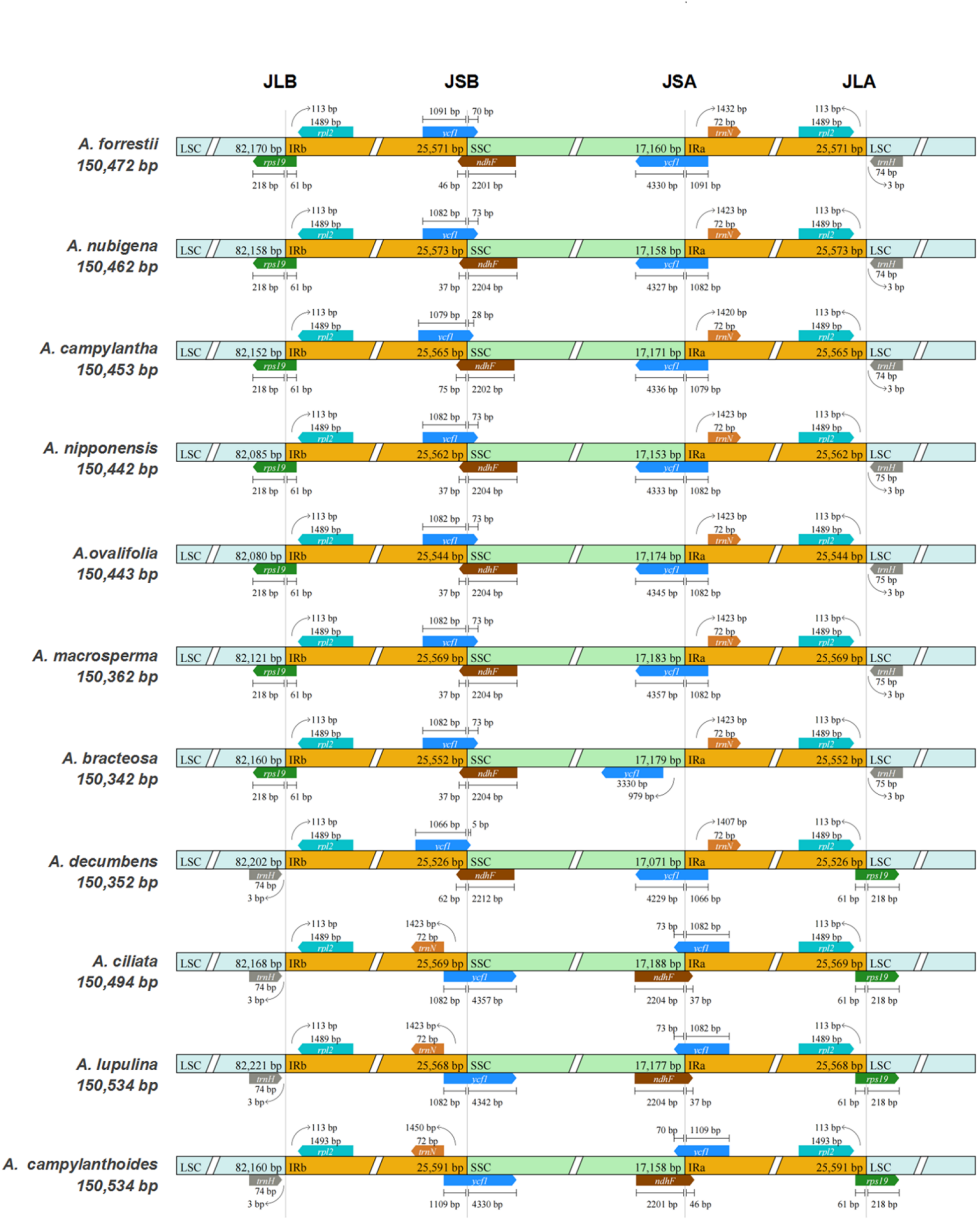
Comparisons of the borders of LSC, SSC, and IRa/b regions among the 11 *Ajuga* plastid genomes.

The expansion/contraction of IR regions is frequently observed during evolution and may account for the disparity in the size of plastid genomes (Menezes et al., 2018). As depicted in **Figure 2**, the *rpl2* gene is entirely situated within the IR regions across all species, while the *trnH* gene exclusively occupies the LSC region, consistent with the cp genomes of most angiosperms (Wang J. et al., 2022; Fang et al., 2023). Besides, the *trnN* gene was entirely located within the IRa region in all species except for *A. ciliata*, *A. lupulina*, and *A. campylanthoides*, where it was utterly localized in the IRb region at varying distances from the junction of SSC/IRb.

Among the seven *Ajuga* species, a truncated copy of *ndhF* genes was identified at the junction of SSC/IRb, starting from SSC and integrating into the IRb region. Conversely, in the remaining species, including *A. ciliata*, *A. lupulina*, and *A. campylanthoides*, *ndhF* was found at the junction of IRa/SSC. In addition, the *ycf1* gene was observed at the IRb/SSC junction in all species. It originated from the IRb region and integrated into SSC, with sizes ranging from 5 to 4,357 bp. Notably, the *ycf1* gene was also present at the IRa/SSC junction in all species except *A. bracteosa*, in which this gene was exclusively present in SSC. This result implied that the *ycf1* could potentially serve as a marker for distinguishing *A. bracteosa.* A previous study also highlighted ycf1 as a powerful barcode for land plants (Dong et al., 2015). Furthermore, the *rps19* gene was consistently located at the boundaries of the IRs/LSC in all *Ajuga* species. This pattern aligns with the cp genomes of other Lamiaceae species, such as *S. mekongensis*, *Mentha spicata*, and *Dracocephalum heterophyllum* (Wu H. et al., 2021; Fu et al., 2022). In summary, the sizes of the cp genomes in the 11 *Ajuga* species vary, and there are noteworthy variations in the junction regions. These findings provide evidence of a distinctive pattern of IR contraction/expansion within the cp genomes of *Ajuga* species, which can be employed to investigate species-specific gene loci.

### 3.2 Codon usage bias of the cp genomes

**Figure 3:**
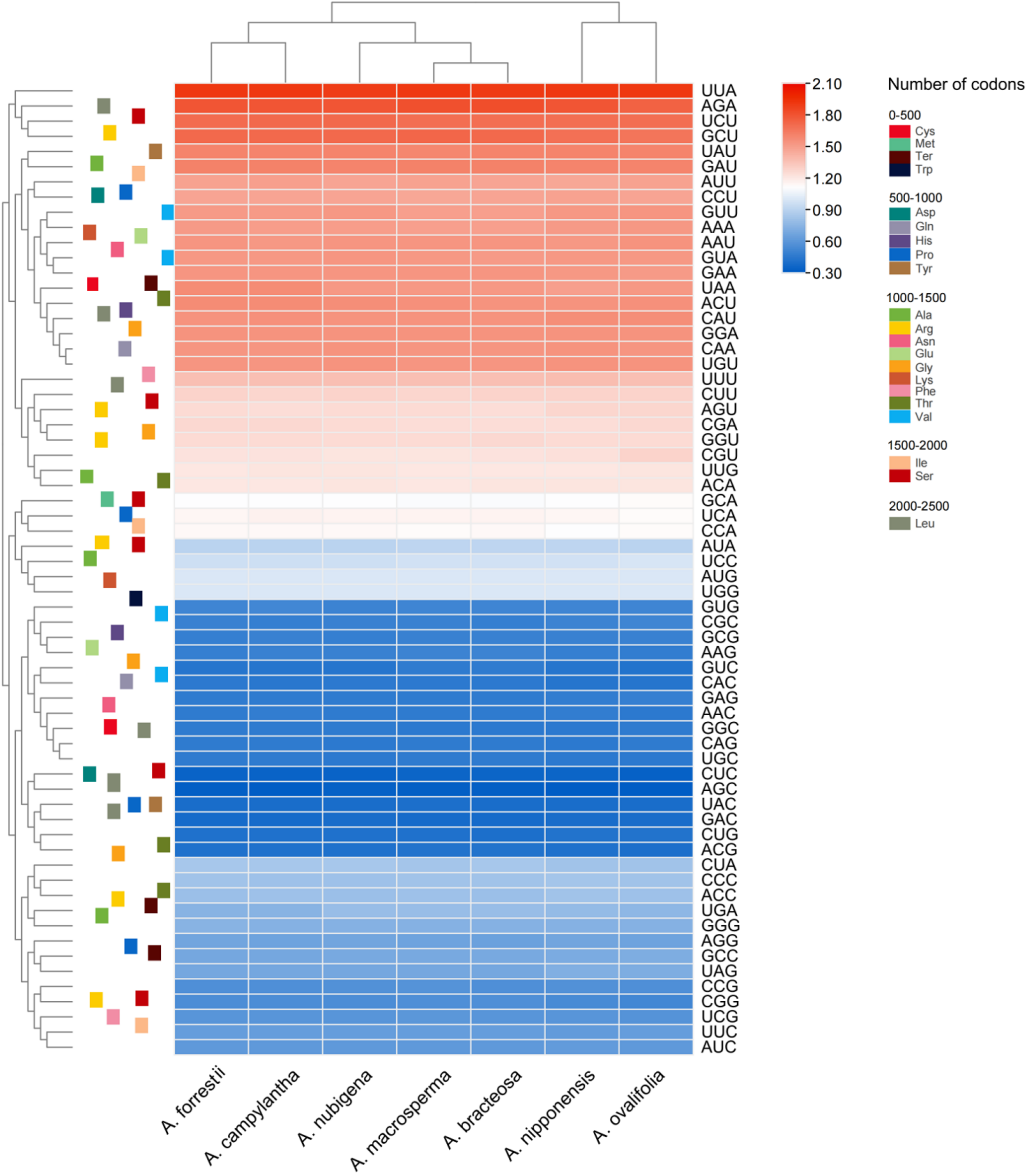
Heat map of the RSCU values among *Ajuga* cp genome.

Analyzing codon usage is essential to evaluate the evolution of the cp genome (Wang N. et al., 2022). In genes of seven *Ajuga* species, 64 codons were identified, of which 61 encoded 24 amino acids. Leucine exhibited the highest frequency among all the amino acids encoded by cp genomes, whereas cysteine was found to be a rare amino acid (**Table S4**). This observation is consistent with the codon usage bias reported by previous studies (Orešič and Shalloway, 1998; Liu et al., 2020). Moreover, pronounced bias towards A or T at the tertiary position of codon was observed, which could be attributed to the high AT proportion in plastid genomes. Similar results were observed in other angiosperm taxa (Wu L. et al., 2021; Jiang et al., 2022). As shown in **Figure 3**, UUA had the highest frequency, followed by AGA, while GCA had the lowest frequency. In *Ajuga*, RSCU values of 30 codons were higher than 1.00, 32 codons had values below 1.00, and two had values of 1.00. An RSCU value below 1.0 suggests that the codon usage frequency is less than expected, while an RSCU value over 1.0 means that the codon usage frequency is more than expected (Ma et al., 2015; Parvathy et al., 2022). This variability in RSCU values reflects evolutionary information resulting from mutation and selection, which is essential in studying organismal evolution (Morton, 2003).

Additionally, the GC proportion of the GC3s was closely associated with codon bias and was an important parameter for evaluating the codon use pattern (Ma et al., 2015; Parvathy et al., 2022). In *Ajuga*, GC3s values ranged from 26.9% to 27.0%, indicating that the genus *Ajuga* preferred A/U ending codons (Zhang et al., 2022). Previous studies have highlighted that high AT content is the primary reason for synonymous codons ending in A/U, potentially linked to natural selection and mutation during evolution (Zhang et al., 2018). Additionally, the Enc varied between 49.80% and 49.94%, while the CAI and the optimal frequency were lower than 0.5. These results suggested a slight bias in codon usage within the seven *Ajuga* taxa.

### 3.3 Repeat sequences

**Figure 4:**
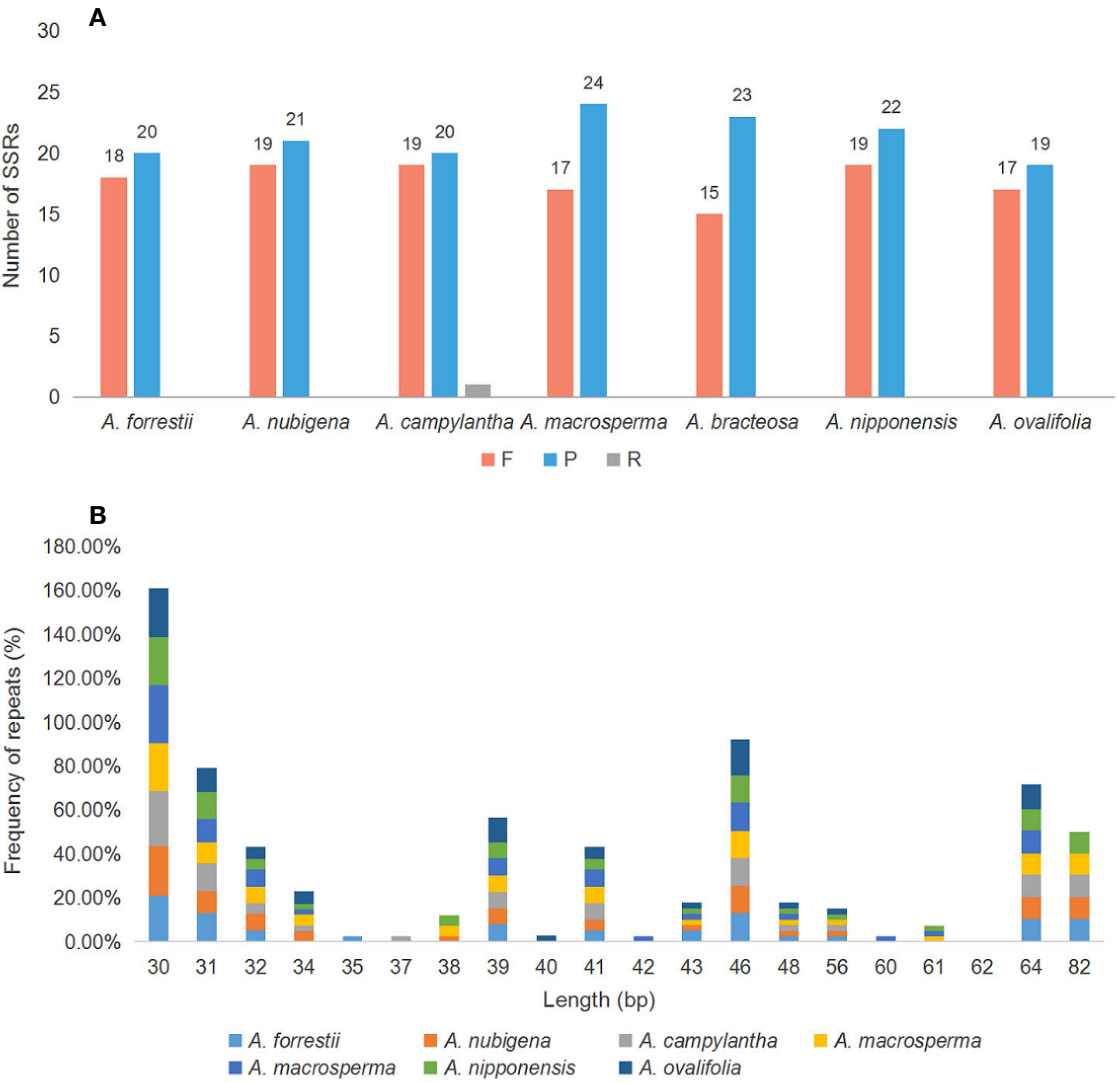
Number of long repetitive repeats on the cp genome of seven *Ajuga* species. **(A)** the number of repeat types (P- palindromic repeats, F, forward repeats; R, Reverse); **(B)** Frequency of the repeats more than 30 bp long.

Large and complex repeat sequences in the cp genome are potential markers for revealing gene rearrangements and losses during evolution (McDonald et al., 2011; Yi et al., 2013). Analysis of oligonucleotide repeat in seven cp genomes indicated that the number and length of repeat sequences differed among genomes and were distributed randomly, with repeat sequences ranging from 30 to 82 bp and most being within 30 - 46 bp (**Figure 4B**). Meanwhile, 274 long repeats were identified, including 149 P repeats, 124 F repeats, and 1 R repeat, with P and F being more than R and C, a pattern consistent with most plastid genomes of angiosperms (Vu et al., 2020; Luo et al., 2022). R repeats were only present in *A. campylantha*, while C repeats were absent in all seven *Ajuga* species (**Figure 4A**). These findings provide a molecular basis for identifying the *Ajuga* species.

**Figure 5:**
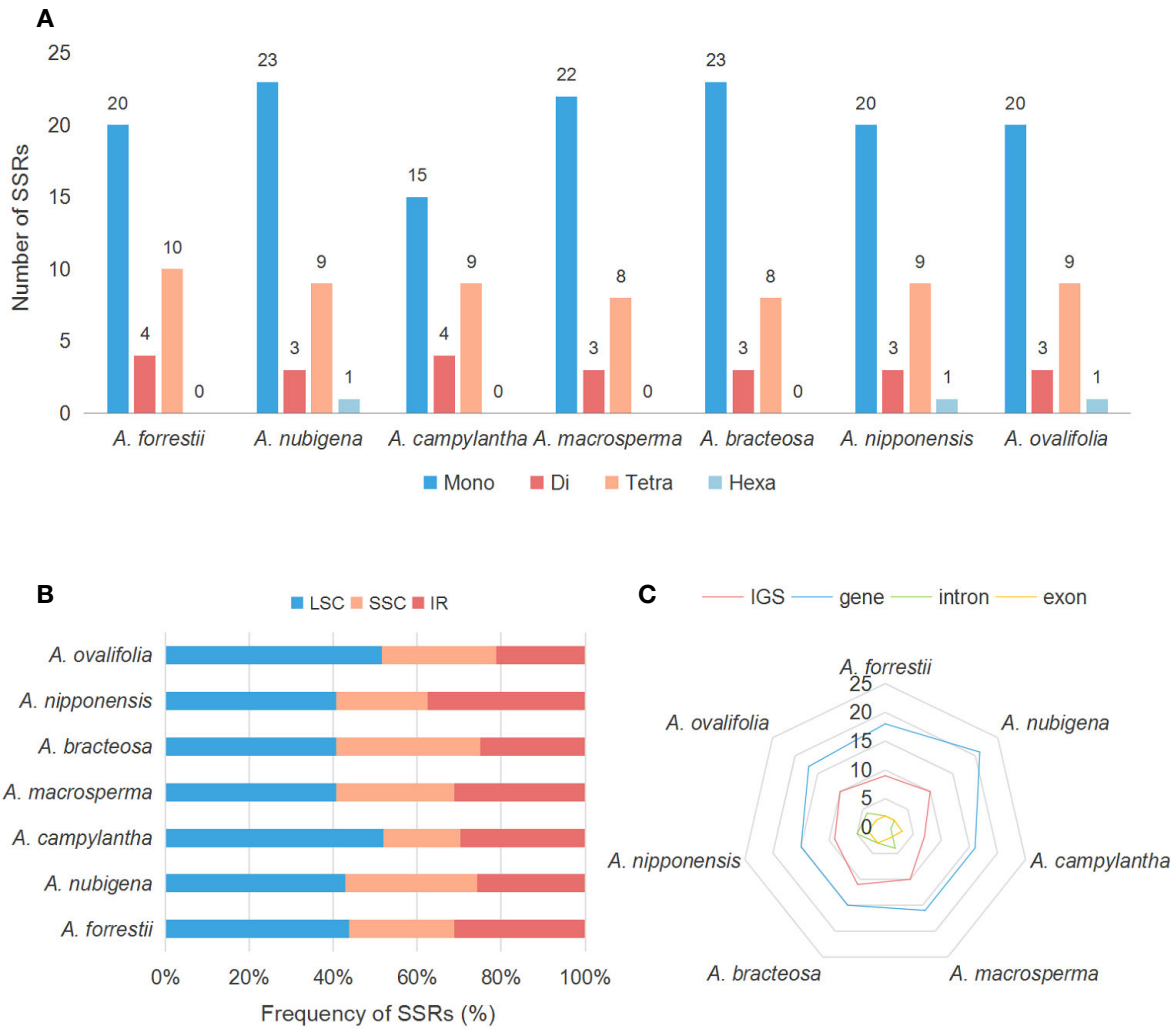
Compares SSR distribution in the cp genomes of seven species. **(A)** the number of different SSR types; **(B)** Frequency of SSRs in the LSC, IR, and SSC region; **(C)** Number of SSRs in the intergenic regions, PCGs, and introns.

SSRs, also known as microsatellites, are popular genetic indicators because of their significant polymorphism, repeatability, and reliability, which can be used to detect genetic diversity, population, and polymorphisms at intraspecific, distant phylogenetic relationships and cultivar levels (Wang et al., 2009; Peng et al., 2022). 34, 36, 28, 33, 34, 33, and 33 SSRs were found in *A. forrestii*, *A. nubigena*, *A. campylantha*, *A. macrosperma*, *A. bracteosa*, *A. nipponensis*, and *A. ovalifolia*, respectively (**Figure 5A**). Among these, mononucleotide (A/T/C) repeats were more than other types of repeats, accounting for almost 62%, as previously reported by Zhou et al. (Zhou et al., 2022). The second most common was tetranucleotide repeat (23.53% - 29.41%), with a predominant motif of AAAG/CTTT and AAAT/ATTT, followed by dinucleotide repeats (8.33% - 14.29%), with a dominant motif of AT/AT. Hexanucleotides (2.78% - 3.03%) were only present in plastomes of *A. nubigena* (AACTAT/AGTTAT) and *A. nipponensis* (AAAAAT/ATTTTT), while trinucleotide or pentanucleotide repeats were absent in all seven *Ajuga* species. These findings indicate that mononucleotide repeats are more frequent than other types, and A/T motifs were the most abundant in the mononucleotide repeats, which is consistent with previous studies (Munyao et al., 2020; Ren et al., 2022). It was suggested that the high amount of mononucleotide repeats in the cp genome may contribute to heritable variations (Bi et al., 2018).

Additionally, the number of SSRs exhibited significant variation across distinct structural and functional regions of the cp genomes (Sun et al., 2022). Frequency analysis revealed that SSRs were more prevalent in LSC (44.55%) than in IR (28.79%) and SSC (26.66%) regions (**Figure 5B**). Besides, most SSRs were located in gene regions and IGS, with an average number of 16 and 9, respectively. In contrast, the regions encompassing introns and exons contained the fewest SSRs, with an average count of 3 and 2, respectively (**Figure 5C**). These findings are consistent with those observed in other Lamiaceae species (Fu et al., 2022). Previous research has emphasized the suitability of SSRs as a genetic marker in plant molecular studies (Khan et al., 2019), particularly in non-coding regions exhibiting high intraspecific variation (Eguiluz et al., 2017). Our investigation indicated that most SSRs were situated within non-coding regions, with a limited presence in the coding areas. Consequently, these SSRs have the potential to serve as markers for discerning various evolutionary changes, such as genetic diversity, and they may even facilitate species differentiation within *Ajuga*.

### 3.4 Comparing genomes and nucleotide diversity

**Figure 6:**
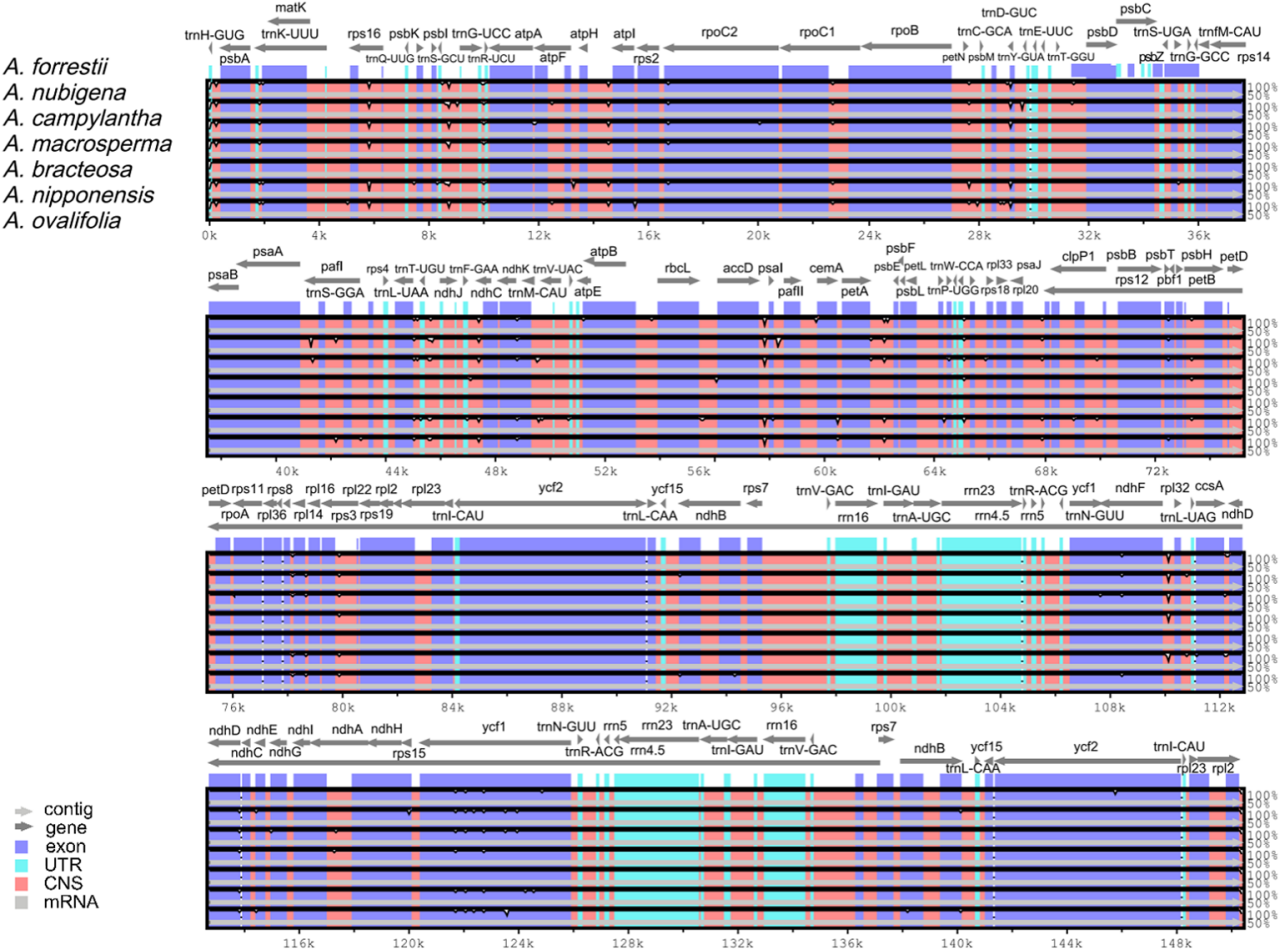
Global comparison of complete genomes of *Ajuga*.

The comparative analysis of cp genomes is a practical approach to investigating the genetic structure and phylogenetic kinships of plants (Daniell et al., 2016). Overall sequence variation in plastid genomes of *Ajuga* indicated a high level of conservation, with the protein-coding regions exhibiting more significant conservation than non-coding regions, except *ndhF, ycf1*, and *ycf2* genes (**Figure 6**). This observation aligns with a previous report on the cp genome of *S. miltiorrhiza* within the Lamiaceae family (Qian et al., 2013). Besides, the most significant discrepancy was mainly found in IGS, e.g., *trnH*(*GUG*)*-psbA, rps16-trnQ*(*UUG*)*, atpH-atpI, rbcL-accD, ndhC-trnV*(*UAC*)*, accD-psaI, trnF*(*GAA*)*-ndhJ*, and *ndhF-rpl32*. Previous studies have identified IGS as hotspots with numerous nucleotide substitutions and indel mutations, making them valuable markers with high resolution for phylogenetic analyses (Drummond, 2008). For example, *rps16-trnQ*(*UUG*) is highly variable in most plants and has been utilized for DNA barcoding in phylogenetic studies across various angiosperm genera (Dong et al., 2012). Similarly, *rbcL-accD* and *trnH*(*GUG*)*-psbA* have been proposed as critical molecular markers for phylogenetic analyses of *Viola* species (Cao et al., 2022). Consequently, these highly variable regions are expected to offer ample genetic information for conducting studies on species delimitation and the phylogenetic evolution of *Ajuga*.

**Figure 7:**
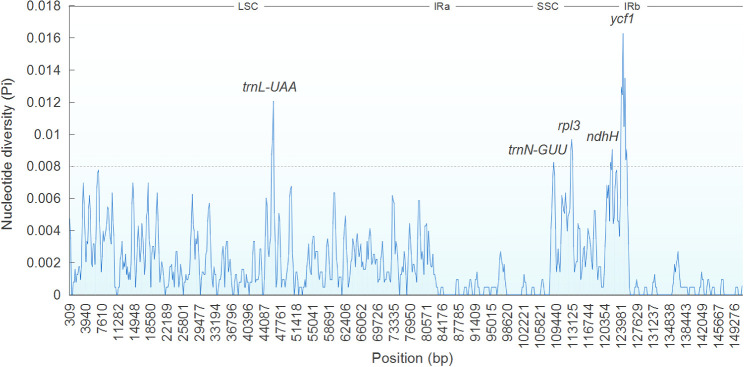
Sliding window analysis of *Ajuga* cp genome.

Sliding window analysis showed that Pi ranged between 0 and 0.01627 across seven *Ajuga* cp genomes examined. Notably, five regions, *trnL-UAA*, *trnN-GUU*, *rpl32*, *ndhH*, and *ycf1*, exhibited higher Pi of > 0.008 (**Figure 7**). Among these genes, the *trnN-GUU* gene displayed the lowest divergence value (0.00825), whereas the *ycf1* regions had the highest (0.01627). Four genes were in SSC regions, and one was in LSC, suggesting that the LSC and SSC regions displayed higher divergence rates than IR regions, which agrees with previous results (Jiang et al., 2022). Based on our findings, we propose that 13 highly variable sites (*atpH-atpI, accD-psaI, trnH(GUG)-psbA, rbcL-accD*, *trnF*(*GAA*)*-ndhJ*, *rps16-trnQ*(*UUG*), *ndhC-trnV*(*UAC*), *ndhF-rpl32*, *trnL-UAA*, *trnN-GUU*, *rpl32*, *ndhH*, and *ycf1*) could serve as potential molecular markers to differentiate *A. bracteosa* and its dopants.

### 3.5 IGS-based species authentication

IGS is often employed as indicators in phylogeny inference at various taxonomic hierarchies, which is more variable and can offer more evolutionarily revealing characters (Fang et al., 2023). Previous molecular studies of the genus *Pogostemon* (Zhang et al., 2020) and *Clerodendrum* (Chen et al., 2023) have demonstrated the high identification capabilities of cp genetic markers. In this study, eight IGSs were extracted from seven *Ajuga* species. ML analyses were conducted on each IGS using IQtree (Nguyen et al., 2015). As illustrated in **Figure S2.1**-**2.8**, the results showed that *A. bracteosa* could be differentiated from its common adulterants based on *ndhF-rpl32, ndhC-trnV(UAC), accD-psaI*, *atpH-atpI*, and *trnH(GUG)-psbA*, while the remaining IGSs were incapable with weak bootstrap values (<70). Previous studies have also identified the *ndhF-rpl32* region as a useful molecular marker for distinguishing *Magnolia polytepala* and its closely related species (Sun et al., 2020) and *ndhC-trnV(UAC)* for distinguishing *Isodon rubescens* and its adulterants (Zhou et al., 2022). Moreover, *accD-psaI* or *atpH-atpI* were probable markers for identifying additional species (Alzahrani, 2021; Zheng et al., 2022).

Although general DNA barcodes (e.g., ITS) can distinguish *A. ciliata* and related taxa (Han et al., 2008), some common adulterants were not investigated. Our results revealed that the studied IGSs exhibited more variability than ITS. The ML tree was constructed based on five IGSs that could differentiate *A. bracteosa* from its common adulterants. The tree demonstrated that all *A. bracteosa* species formed a monophyletic clade, and *A. macrosperma* formed independent branches. Strong support was observed for a sister relationship between *A. macrosperma* and *A. bracteosa* (**Figure S3**). These findings indicate that five combining IGSs can successfully distinguish *A. bracteosa* and its frequent dopants.

Additionally, we designed primers for five IGS and conducted amplification and sequencing experiments (**Table 2**). It is worth noting that due to the lack of residual DNA in three *Ajuga* samples, our supplementary investigation was limited to four species with remaining DNA (*A. bracteosa*, *A. forrestii*, *A. macrosperma*, and *A. campylantha*). The results demonstrated that except for the *trnH*(*GUG*)*-psbA* primers, the other four fragments (*accD-psaI*, *atpH-atpI*, *ndhC-trnV*(*UAC*), and *ndhF-rpl23*) produced products of the expected sizes in the selected *Ajuga* species (refer to **Figure S4**). Both amplification and sequencing achieved a 100% success rate. The sequence data from the four *Ajuga* species align with the cp genome results, with each species exhibiting distinct base differences (refer to **Figure S5.1-5.4**). These findings confirm that the four DNA barcodes are ideal tools for distinguishing *A. bracteosa* from its adulterants.

**Table 2:** Primer design by SnapGene.

Fragment	Primer ID	Base sequence (5’-3′)	Length/bp	Tm/°C	GC/%
*accD-psaI*	accD-psaI-F	GGAGTTTTCTTTGGTGACCT	20	55	45
	accD-psaI-R	AAGGGGTACCTCGATTTACT	20	54	45
*atpH-atpI*	atpH-atpI-F	ACCGTAAAGTAGAAGCGC	18	55	50
	atpH-atpI-R	ATGTATGTGCGACCCAAG	18	55	50
*ndhC-trnV*(*UAC*)	ndhC-trnV(UAC)-2F	ACGCACTCCTATGAACGT	18	56	50
	ndhC-trnV(UAC)-2R	CCTGTCCACAATCAAGGG	18	55	56
*ndhF-rpl32*	ndhF-rpl32-F	TAATTGTTTCCGATTCACCGG	21	55	43
	ndhF-rpl32-R	TTCATTGGTATAGCTGGATGTG	22	55	41
*trnH*(*GUG*)*-psbA*	trnH(GUG)-psbA-F	ATCAAGGCAGTGGATTGTG	19	55	47
	trnH(GUG)-psbA-R	AAGAGGGGTTATTGCTCCT	19	55	47

### 3.6 Phylogenetic analysis

**Figure 8:**
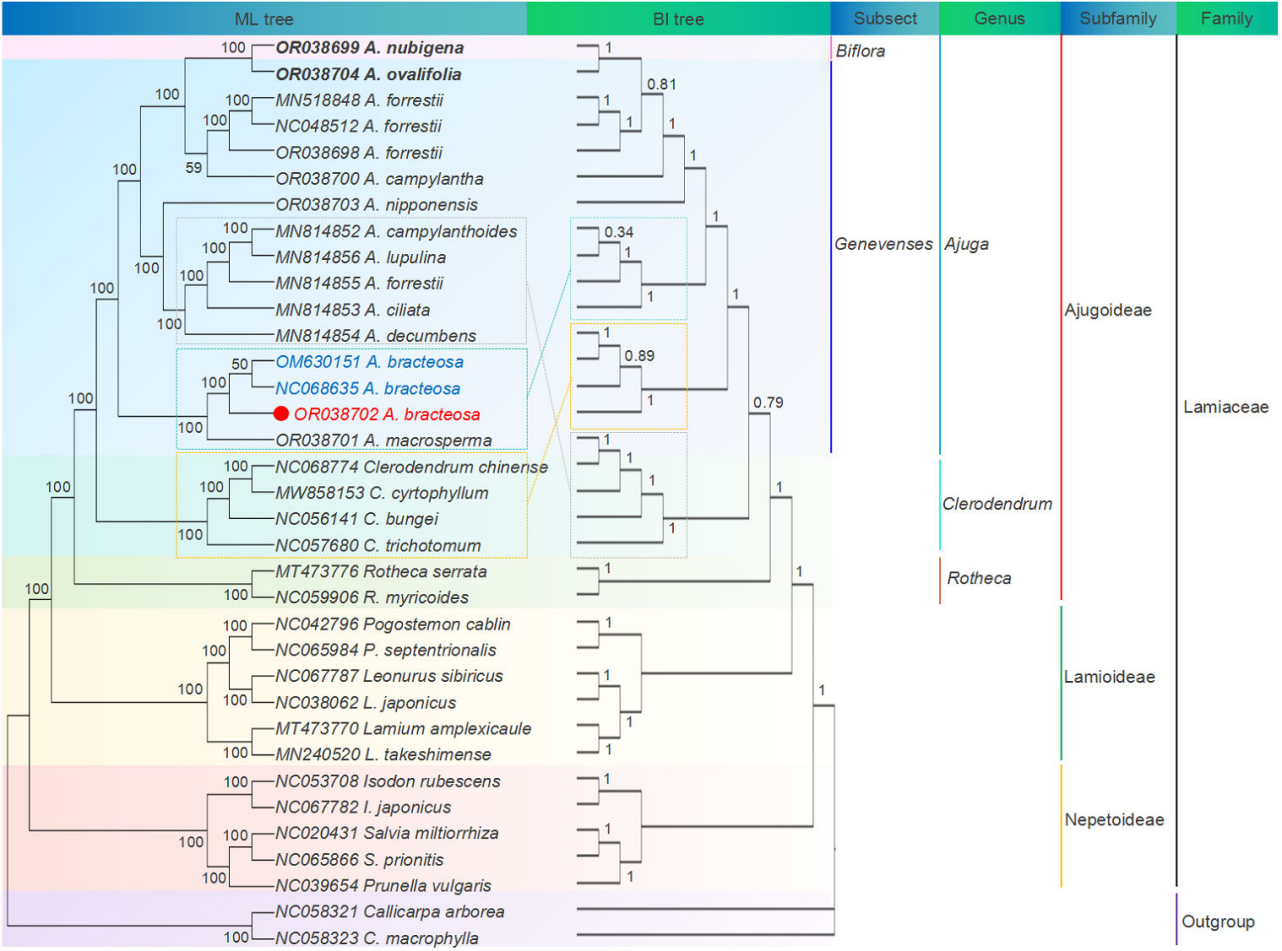
ML and BI phylogenetic tree based on 68 common PCGs of 35 species.

Cp genomes have a wealth of phylogenetic information and are extensively employed for reconstructing phylogenies and conducting plant population analyses (Yan et al., 2019). Here, ML and BI trees were reconstructed using cp genomes and common PCGs of 35 species, respectively. **Figure 8** illustrates ML and BI trees based on 68 shared PCGs, showing similar topologies. Among the three subfamilies, Ajugoideae and Lamioideae Harley emerged as sister taxa, while Nepetoideae (Dumort.) Burnett appeared as the sister group to the clade consisting of Ajugoideae and Lamioideae. The tree’s crown was occupied by the subfamily Ajugoideae, which included the genera *Ajuga, Clerodendrum*, and *Rotheca*. These results approve *Ajuga*’s position within the Lamiaceae family and align with previous phylogenomic results (Tao et al., 2019). Additionally, the ML tree based on PCGs showed that genus *Ajuga* was divided into triple clades: (i) clade A included *A. nubigena*, *A. ovalifolia*, *A. forrestii* (Genbank MN518848.1), *A. forrestii* (Genbank NC048512.1), *A. forrestii* (Genbank OR038698)*, A. campylantha*, and *A. nipponensis*; (ii) clade B included *A. bracteosa* (Genbank M630151.1), *A. bracteosa* (Genbank NC068635.1), *A. bracteosa* (Genbank OR038702), and *A. macrosperma*; (iii) clade C included *A. ciliata*, *A. forrestii*, *A. campylanthoides*, *A. decumbens*, and *A. lupulina.*

Phylogenetic analysis also demonstrated that all *A. bracteosa* species formed a monophyletic clade. *A. bracteosa* (GenBank OR038702) was an independent branch of the phylogeny and deeply nested within clade B, with strong support (BS = 100) for the sister relationship between *A. bracteosa* and *A. macrosperma*, suggesting that the cp genome could distinguish *A. bracteosa* from other species. Notably, the samples of *A. forrestii* (Genbank MN814855.1) did not form a monophyletic group and were placed in different branches from three individuals of *A. forrestii*. Previous studies also confirmed that intraspecific diversity existed in *Artemisia argyi* (Chen et al., 2022), *Isodon rubescens* (Zhou et al., 2022), and *Phyllanthus urinaria* collected from different geographical areas (Fang et al., 2023). This phenomenon could potentially be attributed to the influence of the geographical area of origin on the variation in *A. forrestii*.

Additionally, a strong support value of 100 was observed for the sister relationship between *A. nubigena* from Subsect. Biflora and *A. ovalifolia* from Subsect. Genevenses, contradicting the taxonomic findings presented in Flora of China (FOC) (Li and Hedge, 1994). The BI tree also confirmed the same result with the posterior probabilities value of 1. This finding suggests a potential limitation in the current classification of Subsect. Biflora as independent entities. Therefore, based on this evidence, we propose an alternative classification scheme that amalgamates Subsect. Biflorae and Subsect. Genevenses into a single group rather than maintaining separate categorizations.

Notably, *Clerodendreae* is classified as part of the *Verbenaceae* family in the FOC (Chen and Gilbert, 1994). Our study confirms its placement within the subfamily Ajugoideae, aligning with the APG IV classification (Angiospermm, 2016). Therefore, we recommend reclassifying the genus *Clerodendreae* under Lamiaceae.

Furthermore, the phylogenetic trees constructed using cp genomes (**Figure S6.1-6.2**) and common PCGs (**Figure 8** and **Figure S6.3**) showed a high degree of similarity. Previous studies have suggested that using cp genomes may lead to missing relationships due to length variations, gaps/index deletions, and inappropriate models of DNA evolution in concatenated datasets (Goremykin et al., 2005; Lockhart and Penny, 2005). Given that the genetic divergence in gene-encoding regions occurs more slowly than in non-coding sequences, we considered that utilization of common PCGs is more appropriate for the identification and phylogeny analysis of *Ajuga*.

In conclusion, our findings serve as a valuable basis and reference for utilizing plastid genomes and common PCGs in species identification, contributing to a better understanding of *Ajuga*’s phylogeny.

### 3.7 Divergence time

**Figure 9:**
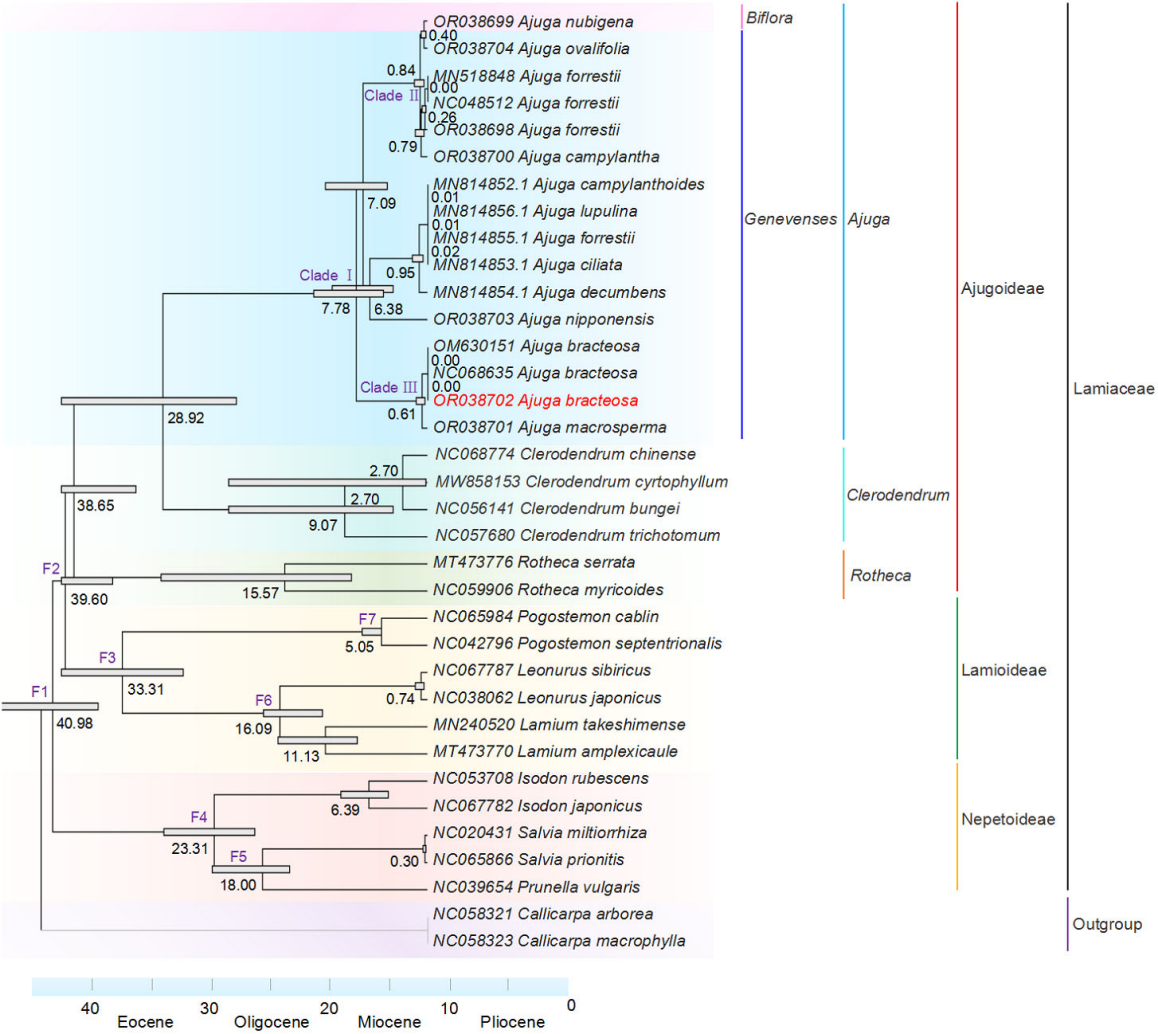
Divergence times estimation based on cp genomes.

The 35 cp genomes from Lamiaceae family plants, including 16 *Ajuga* species, were utilized to estimate the divergence time based on the ML tree. As shown in **Figure 9**, the subfamilies of Ajugoideae and Lammiodeae shared a common ancestor in the late Eocene (39.60 Mya), and the split between *Ajuga, Clerodendrum*, and *Rotheca* could occur in the Oligocene (38.65 Mya). The process of speciation of the *Ajuga* genus was estimated to originate at 7.78 Mya in the late Miocene. Notably, the Three main lineages (clade I: 6.38 Mya; clade II: 0.84 Mya; clade III: 0.61 Mya) within the *Ajuga* genus diverged in the late Miocene and continued throughout the Pleistocene. This time frame aligns with the final stages of rapid uplift in the Qinghai-Tibetan Plateau (QTP), which has been recognized as a region abundant in biodiversity and a source of various herbal resources (Wen et al., 2014; Favre et al., 2015). Biasatti et al. also suggested that QTP could have influenced the geographical environment, climate, and distribution/divergence of species (Biasatti et al., 2012). These findings imply that the interspecific divergence of the *Ajuga* species may have a close association with the uplift of the QTP.

In addition, the Pleistocene has been proposed as a critical period for refugial isolation and subsequent lineage formation, leading to modern species diversity (Johnson and Cicero, 2004). According to the Pleistocene speciation model, glacial cycles during this era acted as a ‘species pump,’ contributing significantly to the diversity of organisms inhabiting temperate regions (Pyron and Burbrink, 2009). Previous research has also indicated that the extreme climatic fluctuations of the Pleistocene played a pivotal role in driving diversification between lineages in specific taxa (Hewitt, 1999; Bryson et al., 2012).

Consequently, it is postulated that the intense uplift of the QTP, coupled with the climatic oscillations of the Pleistocene, may have influenced diversification and facilitated the radiation of *Ajuga* species.

## 4 Conclusions

In this study, plastid genomes of seven *Ajuga* species were *de novo* assembled based on short sequencing reads, and cp genome sequences of *A. macrosperma* and *A. ovalifolia* were reported for the first time. These plastid genomes were broadly conserved, displaying comparable gene organization and content. We demonstrated the utility of PCGs integration in phylogeny investigations of *Ajuga.* Phylogeny analysis of 68 common PCGs strongly supported the taxonomic placement of *Ajuga* within the Lamiaceae family. It explicitly supported a sister relationship between *A. nubigena* from Subsect. Biflora and *A. ovalifolia* from Subsect. Genevense. Consequently, we propose amalgamating Subsect. Biflorae and Subsect. Genevenses into a single group, advocating against their separate categorization. Four highly variable cp loci, including *atpH-atpI*, *accD-psaI*, *ndhC-trnV*(*UAC*), and *ndhF-rpl23*, were identified, which hold promise as markers for distinguishing *A. bracteosa* from its common adulterants. The divergence time of *Ajuga* occurred in the early Pliocene, possibly due to the intense uplift of QTP and the global cooling event. In summary, this study provides a valued reference for ensuring the efficacy and safety of clinical application while also facilitating bioprospecting and conservation efforts concerning the *Ajuga* species.

## Data availability statement

The datasets presented in this study can be found in online repositories. The names of the repository/repositories and accession number(s) can be found in the article/**Supplementary Material**.

## Author contributions

MS and BD participated in the conception and design of the research. JinW and BL collected the species. JiaW, GD, and JZ are responsible for analyzing and processing data. MS wrote the manuscript. BD and JinW revised this manuscript. All authors contributed to the article and approved the submitted version.
