# Supplementary material for: Comparative analysis of chloroplast genome and new insights into phylogenetic relationships of Ajuga and common adulterants
Source: Front Plant Sci. 2023 Oct 25;14:1251829. doi: 10.3389/fpls.2023.1251829 (PMC10634298; doi:10.3389/fpls.2023.1251829)
Supplement: Supplementary file 1 [file DataSheet_1.pdf]

Table S1 Information about the samples collected

No.	Species	Locality	Voucher specimen
1	*A. forrestii*	Kunming, Yunnan, China	YN2022JGC01
2	*A. nubigena*	Kunming, Yunnan, China	YN2022JGC02
3	*A. campylantha*	Kunming, Yunnan, China	YN2022JGC03
4	*A. macrosperma*	Kunming, Yunnan, China	YN2022JGC04
5	*A. bracteosa*	Kunming, Yunnan, China	YN2022JGC05
6	*A. nipponensis*	Kunming, Yunnan, China	YN2022JGC06
7	*A. ovalifolia*	Kunming, Yunnan, China	YN2022JGC07

Table S2 Species information downloaded by NCBI

No.	Species	GenBank accession	No.	Species	GenBank accession
1	*Ajuga bracteosa*	NC068635	16	*Lamium amplexicaule*	MT473770
2	*Ajuga bracteosa*	OM630151	17	*Lamium takeshimense*	MN240520
3	*Ajuga lupulina*	MN814856	18	*Isodon rubescens*	NC053708
4	*Ajuga forrestii*	MN814855	19	*Isodon japonicus*	NC067782
5	*Ajuga forrestii*	NC048512	20	*Salvia prionitis*	NC065866
6	*Ajuga forrestii*	MN518848	21	*Salvia miltiorrhiza*	NC020431
7	*Ajuga campylanthoides*	MN814852	22	*Prunella vulgaris*	NC039654
8	*Ajuga decumbens*	MN814854	23	*Callicarpa macrophylla*	NC058323
9	*Ajuga ciliata*	MN814853	24	*Callicarpa arborea*	NC058321
10	*Rotheca serrata*	MT473776	25	*Clerodendrum chinense*	NC068774
11	*Rotheca myricoides*	NC059906	26	*Clerodendrum cyrtophyllum*	MW858153
12	*Pogostemon septentrionalis*	NC065984	27	*Clerodendrum bungei*	NC056141
13	*Pogostemon cablin*	NC042796	28	*Clerodendrum trichotomum*	NC057680
14	*Leonurus sibiricus*	NC067787			
15	*Leonurus japonicus*	NC038062			

Table S3 Gene composition in *Ajuga* cp genome

Category for genes	Group of genes	Name of genes
Self replication	Ribosomal RNAs	*rrn16S*(×2), *rrn23S*(×2), *rrn4.5S*(×2), *rrn5S*(×2)
	Transfer RNAs	*trnH-GUG, trnK-UUU*, trnQ-UUG, trnS-GCU, trnG-UCC*, trnR-UCU, trnC-GCA, trnD-GUC, trnY-GUA, trnE-UUC, trnT-GGU, trnS-UGA, trnG-GCC, trnS-GGA, trnT-UGU, trnL-UAA*, trnV-UAC*, trnM-CAU, trnW-CCA, trnP-UGG, trnI-CAU*(×2), *trnL-CAA*(×2), *trnV-GAC*(×2), *trnI-GAU*(×2)*, *trnA-UGC*(×2)*, *trnR-ACG*(×2), *trnN-GUU*(×2), *trnL-UAG*
	Small subunit of ribosome	*rps11, rps12*(×2), *rps14, rps15, rps16*, rps18, rps19, rps2, rps3, rps4, rps7*(×2), *rps8*
	Large subunit of ribosome	*rpl14, rpl16*, rpl2*(×2)*, *rpl20, rpl22, rpl23, rpl23, rpl32, rpl33, rpl36*
	RNA polymerase	*rpoA, rpoB, rpoC1*, rpoC2*
Genes for photosynthesis	Photosystem I	*psaA, psaB, psaC, psaI, psaJ*
	Photosystem II	*psbA, psbB, psbC, psbD, psbE, psbF, psbI, psbJ, psbK, psbL, psbM, psbN, psbT, psbZ, ycf3***
	Cytochrome b/f complex	*petA, petB*, petD*, petG, petL, petN*
	ATP synthase	*atpA, atpB, atpE, atpF*, atpH, atpI*
	Rubisco	*rbcL*
	NADH dehydrogenase	*ndhA*, ndhB*(×2)*, *ndhC, ndhD, ndhE, ndhF, ndhG, ndhH, ndhI, ndhJ, ndhK*
Other genes	Maturase	*matK*
	Envelop membrane protein	*cemA*
	acetyl-CoA-carboxylase	*accD*
	c-type cytochrome synthesis	*ccsA*
	Protease	*clpP***
Genes of unknown function	Conserved open reading frames	*ycf1, ycf2*(×2), *ycf4*

Intron-containing genes are marked by asterisks (*)

Table S4.1 Codons in the cp genome of *A. forrestii*

Amino acid	Codon	No.	RSCU	Amino acid	Codon	No.	RSCU	Amino acid	Codon	No.	RSCU	Amino acid	Codon	No.	RSCU
Phe	UUU	833	1.37	Ser	UCU	458	1.71	Tyr	UAU	627	1.61	Cys	UGU	179	1.54
	UUC	379	0.63		UCC	258	0.96		UAC	153	0.39		UGC	53	0.46
Leu	UUA	722	1.92		UCA	309	1.15	TER	UAA	28	1.58	TER	UGA	13	0.74
	UUG	454	1.21		UCG	157	0.59		UAG	12	0.68	Trp	UGG	385	1
	CUU	483	1.29	Pro	CCU	334	1.48	His	CAU	389	1.55	Arg	CGU	263	1.23
	CUC	127	0.34		CCC	184	0.82		CAC	113	0.45		CGC	104	0.49
	CUA	314	0.84		CCA	256	1.14	Gln	CAA	589	1.54		CGA	272	1.27
	CUG	153	0.41		CCG	127	0.56		CAG	175	0.46		CGG	117	0.55
Ile	AUU	886	1.49	Thr	ACU	419	1.58	Asn	AAU	766	1.54	Ser	AGU	338	1.26
	AUC	364	0.61		ACC	213	0.8		AAC	232	0.46		AGC	86	0.32
	AUA	539	0.9		ACA	322	1.21	Lys	AAA	851	1.52	Arg	AGA	384	1.8
Met	AUG	488	1		ACG	108	0.41		AAG	272	0.48		AGG	143	0.67
Val	GUU	444	1.52	Ala	GCU	511	1.72	Asp	GAU	698	1.62	Gly	GGU	466	1.26
	GUC	126	0.43		GCC	203	0.68		GAC	165	0.38		GGC	169	0.46
	GUA	447	1.53		GCA	329	1.11	Glu	GAA	855	1.54		GGA	574	1.55
	GUG	150	0.51		GCG	143	0.48		GAG	258	0.46		GGG	273	0.74

Table S4.2 Codons in the cp genome of *A. nubigena*

Amino acid	Codon	No.	RSCU	Amino acid	Codon	No.	RSCU	Amino acid	Codon	No.	RSCU	Amino acid	Codon	No.	RSCU
Phe	UUU	831	1.37	Ser	UCU	459	1.72	Tyr	UAU	633	1.61	Cys	UGU	179	1.55
	UUC	380	0.63		UCC	257	0.96		UAC	153	0.39		UGC	52	0.45
Leu	UUA	715	1.9		UCA	309	1.16	TER	UAA	27	1.53	TER	UGA	14	0.79
	UUG	458	1.22		UCG	157	0.59		UAG	12	0.68	Trp	UGG	386	1
	CUU	486	1.29	Pro	CCU	335	1.48	His	CAU	391	1.55	Arg	CGU	263	1.22
	CUC	129	0.34		CCC	184	0.81		CAC	112	0.45		CGC	106	0.49
	CUA	314	0.84		CCA	257	1.14	Gln	CAA	592	1.55		CGA	272	1.26
	CUG	154	0.41		CCG	129	0.57		CAG	174	0.45		CGG	119	0.55
Ile	AUU	885	1.48	Thr	ACU	419	1.57	Asn	AAU	768	1.54	Ser	AGU	336	1.26
	AUC	365	0.61		ACC	214	0.8		AAC	229	0.46		AGC	87	0.33
	AUA	539	0.9		ACA	325	1.22	Lys	AAA	848	1.51	Arg	AGA	387	1.8
Met	AUG	490	1		ACG	109	0.41		AAG	272	0.49		AGG	145	0.67
Val	GUU	448	1.53	Ala	GCU	513	1.72	Asp	GAU	693	1.61	Gly	GGU	466	1.26
	GUC	125	0.43		GCC	205	0.69		GAC	168	0.39		GGC	172	0.46
	GUA	453	1.54		GCA	333	1.11	Glu	GAA	864	1.54		GGA	576	1.55
	GUG	148	0.5		GCG	145	0.48		GAG	261	0.46		GGG	271	0.73

Table S4.3 Codons in the cp genome of *A. campylantha*

Amino acid	Codon	No.	RSCU	Amino acid	Codon	No.	RSCU	Amino acid	Codon	No.	RSCU	Amino acid	Codon	No.	RSCU
Phe	UUU	831	1.37	Ser	UCU	458	1.72	Tyr	UAU	629	1.61	Cys	UGU	179	1.54
	UUC	382	0.63		UCC	256	0.96		UAC	151	0.39		UGC	54	0.46
Leu	UUA	721	1.92		UCA	313	1.17	TER	UAA	28	1.58	TER	UGA	13	0.74
	UUG	459	1.22		UCG	157	0.59		UAG	12	0.68	Trp	UGG	385	1
	CUU	481	1.28	Pro	CCU	335	1.48	His	CAU	389	1.56	Arg	CGU	265	1.23
	CUC	128	0.34		CCC	184	0.81		CAC	109	0.44		CGC	104	0.48
	CUA	314	0.84		CCA	258	1.14	Gln	CAA	587	1.54		CGA	273	1.27
	CUG	153	0.41		CCG	127	0.56		CAG	176	0.46		CGG	118	0.55
Ile	AUU	889	1.5	Thr	ACU	420	1.58	Asn	AAU	768	1.53	Ser	AGU	334	1.25
	AUC	360	0.61		ACC	215	0.81		AAC	235	0.47		AGC	84	0.31
	AUA	533	0.9		ACA	323	1.21	Lys	AAA	848	1.51	Arg	AGA	388	1.8
Met	AUG	486	1		ACG	108	0.41		AAG	272	0.49		AGG	143	0.66
Val	GUU	444	1.52	Ala	GCU	512	1.72	Asp	GAU	698	1.62	Gly	GGU	466	1.26
	GUC	127	0.43		GCC	201	0.68		GAC	165	0.38		GGC	170	0.46
	GUA	447	1.53		GCA	330	1.11	Glu	GAA	855	1.54		GGA	575	1.55
	GUG	151	0.52		GCG	145	0.49		GAG	257	0.46		GGG	270	0.73

Table S4.4 Codons in the cp genome of *A. macrosperma*

Amino acid	Codon	No.	RSCU	Amino acid	Codon	No.	RSCU	Amino acid	Codon	No.	RSCU	Amino acid	Codon	No.	RSCU
Phe	UUU	846	1.37	Ser	UCU	459	1.7	Tyr	UAU	633	1.6	Cys	UGU	180	1.54
	UUC	386	0.63		UCC	262	0.97		UAC	156	0.4		UGC	54	0.46
Leu	UUA	729	1.92		UCA	314	1.16	TER	UAA	27	1.53	TER	UGA	14	0.79
	UUG	460	1.21		UCG	161	0.6		UAG	12	0.68	Trp	UGG	387	1
	CUU	489	1.28	Pro	CCU	332	1.47	His	CAU	392	1.55	Arg	CGU	265	1.22
	CUC	134	0.35		CCC	184	0.82		CAC	115	0.45		CGC	106	0.49
	CUA	317	0.83		CCA	255	1.13	Gln	CAA	594	1.54		CGA	271	1.25
	CUG	155	0.41		CCG	130	0.58		CAG	177	0.46		CGG	121	0.56
Ile	AUU	895	1.49	Thr	ACU	422	1.56	Asn	AAU	786	1.54	Ser	AGU	340	1.26
	AUC	365	0.61		ACC	218	0.81		AAC	235	0.46		AGC	86	0.32
	AUA	547	0.91		ACA	328	1.21	Lys	AAA	859	1.5	Arg	AGA	395	1.82
Met	AUG	492	1		ACG	114	0.42		AAG	286	0.5		AGG	142	0.66
Val	GUU	440	1.5	Ala	GCU	515	1.73	Asp	GAU	695	1.62	Gly	GGU	470	1.26
	GUC	136	0.46		GCC	205	0.69		GAC	164	0.38		GGC	169	0.45
	GUA	447	1.53		GCA	328	1.1	Glu	GAA	879	1.54		GGA	577	1.55
	GUG	148	0.51		GCG	145	0.49		GAG	262	0.46		GGG	271	0.73

Table S4.5 Codons in the cp genome of *A. bracteosa*

Amino acid	Codon	No.	RSCU	Amino acid	Codon	No.	RSCU	Amino acid	Codon	No.	RSCU	Amino acid	Codon	No.	RSCU
Phe	UUU	849	1.37	Ser	UCU	459	1.7	Tyr	UAU	634	1.61	Cys	UGU	180	1.54
	UUC	387	0.63		UCC	263	0.97		UAC	156	0.39		UGC	54	0.46
Leu	UUA	727	1.91		UCA	314	1.16	TER	UAA	27	1.53	TER	UGA	14	0.79
	UUG	460	1.21		UCG	160	0.59		UAG	12	0.68	Trp	UGG	387	1
	CUU	489	1.29	Pro	CCU	332	1.48	His	CAU	393	1.55	Arg	CGU	266	1.23
	CUC	134	0.35		CCC	185	0.82		CAC	115	0.45		CGC	105	0.48
	CUA	318	0.84		CCA	256	1.14	Gln	CAA	591	1.54		CGA	271	1.25
	CUG	154	0.4		CCG	127	0.56		CAG	177	0.46		CGG	121	0.56
Ile	AUU	893	1.48	Thr	ACU	421	1.55	Asn	AAU	787	1.54	Ser	AGU	339	1.25
	AUC	365	0.61		ACC	218	0.8		AAC	236	0.46		AGC	86	0.32
	AUA	547	0.91		ACA	330	1.22	Lys	AAA	861	1.5	Arg	AGA	397	1.83
Met	AUG	493	1		ACG	115	0.42		AAG	285	0.5		AGG	142	0.65
Val	GUU	440	1.5	Ala	GCU	514	1.72	Asp	GAU	691	1.62	Gly	GGU	470	1.27
	GUC	136	0.46		GCC	205	0.69		GAC	163	0.38		GGC	169	0.45
	GUA	446	1.52		GCA	328	1.1	Glu	GAA	878	1.54		GGA	577	1.55
	GUG	148	0.51		GCG	146	0.49		GAG	263	0.46		GGG	270	0.73

Table S4.6 Codons in the cp genome of *A. nipponensis*

Amino acid	Codon	No.	RSCU	Amino acid	Codon	No.	RSCU	Amino acid	Codon	No.	RSCU	Amino acid	Codon	No.	RSCU
Phe	UUU	831	1.38	Ser	UCU	458	1.7	Tyr	UAU	638	1.61	Cys	UGU	181	1.55
	UUC	377	0.62		UCC	262	0.97		UAC	156	0.39		UGC	53	0.45
Leu	UUA	727	1.92		UCA	311	1.15	TER	UAA	27	1.5	TER	UGA	14	0.78
	UUG	459	1.21		UCG	159	0.59		UAG	13	0.72	Trp	UGG	389	1
	CUU	489	1.29	Pro	CCU	340	1.49	His	CAU	399	1.57	Arg	CGU	271	1.24
	CUC	131	0.35		CCC	189	0.83		CAC	110	0.43		CGC	109	0.5
	CUA	310	0.82		CCA	254	1.11	Gln	CAA	599	1.55		CGA	275	1.25
	CUG	158	0.42		CCG	129	0.57		CAG	176	0.45		CGG	119	0.54
Ile	AUU	892	1.47	Thr	ACU	420	1.55	Asn	AAU	773	1.53	Ser	AGU	345	1.28
	AUC	374	0.62		ACC	222	0.82		AAC	236	0.47		AGC	85	0.31
	AUA	549	0.91		ACA	331	1.22	Lys	AAA	864	1.52	Arg	AGA	395	1.8
Met	AUG	493	1		ACG	111	0.41		AAG	275	0.48		AGG	146	0.67
Val	GUU	455	1.53	Ala	GCU	515	1.69	Asp	GAU	700	1.61	Gly	GGU	481	1.26
	GUC	129	0.43		GCC	214	0.7		GAC	170	0.39		GGC	170	0.45
	GUA	457	1.53		GCA	338	1.11	Glu	GAA	871	1.53		GGA	591	1.55
	GUG	151	0.51		GCG	150	0.49		GAG	265	0.47		GGG	279	0.73

Table S4.7 Codons in the cp genome of *A. ovalifolia*

Amino acid	Codon	No.	RSCU	Amino acid	Codon	No.	RSCU	Amino acid	Codon	No.	RSCU	Amino acid	Codon	No.	RSCU
Phe	UUU	792	1.37	Ser	UCU	445	1.7	Tyr	UAU	621	1.61	Cys	UGU	177	1.55
	UUC	366	0.63		UCC	259	0.99		UAC	149	0.39		UGC	51	0.45
Leu	UUA	708	1.92		UCA	296	1.13	TER	UAA	28	1.53	TER	UGA	14	0.76
	UUG	451	1.22		UCG	152	0.58		UAG	13	0.71	Trp	UGG	382	1
	CUU	474	1.29	Pro	CCU	334	1.48	His	CAU	395	1.57	Arg	CGU	278	1.29
	CUC	121	0.33		CCC	185	0.82		CAC	107	0.43		CGC	109	0.51
	CUA	303	0.82		CCA	255	1.13	Gln	CAA	583	1.54		CGA	274	1.27
	CUG	155	0.42		CCG	126	0.56		CAG	173	0.46		CGG	112	0.52
Ile	AUU	875	1.49	Thr	ACU	409	1.56	Asn	AAU	751	1.55	Ser	AGU	334	1.28
	AUC	361	0.61		ACC	212	0.81		AAC	221	0.45		AGC	82	0.31
	AUA	531	0.9		ACA	320	1.22	Lys	AAA	835	1.53	Arg	AGA	378	1.75
Met	AUG	481	1		ACG	106	0.4		AAG	258	0.47		AGG	142	0.66
Val	GUU	452	1.54	Ala	GCU	509	1.67	Asp	GAU	679	1.61	Gly	GGU	471	1.26
	GUC	124	0.42		GCC	215	0.71		GAC	165	0.39		GGC	172	0.46
	GUA	451	1.53		GCA	345	1.13	Glu	GAA	824	1.52		GGA	581	1.55
	GUG	149	0.51		GCG	150	0.49		GAG	260	0.48		GGG	275	0.73

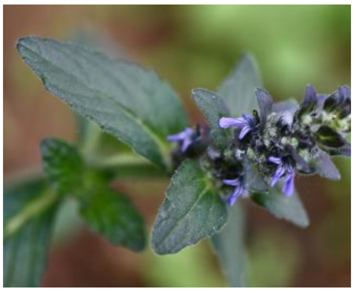

A. forrestii

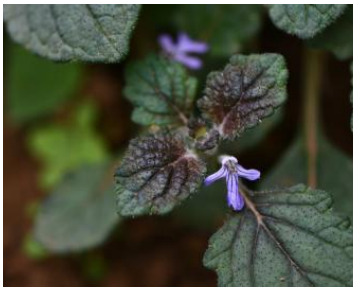

A. nubigena

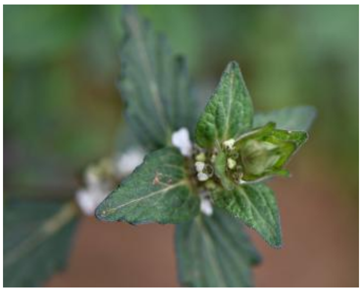

A. campylantha

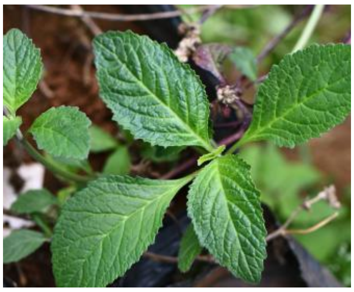

A. macrosperma

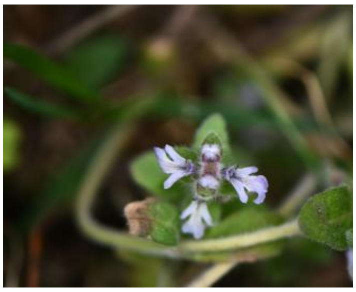

A. bracteosa

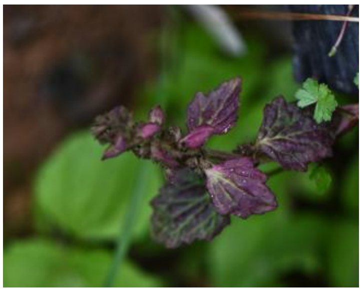

A. nipponensis

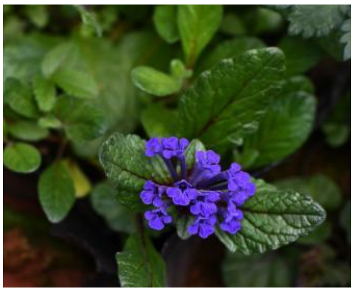

A. ovalifolia

Figure S1 Pictures of *Ajuga* plants.

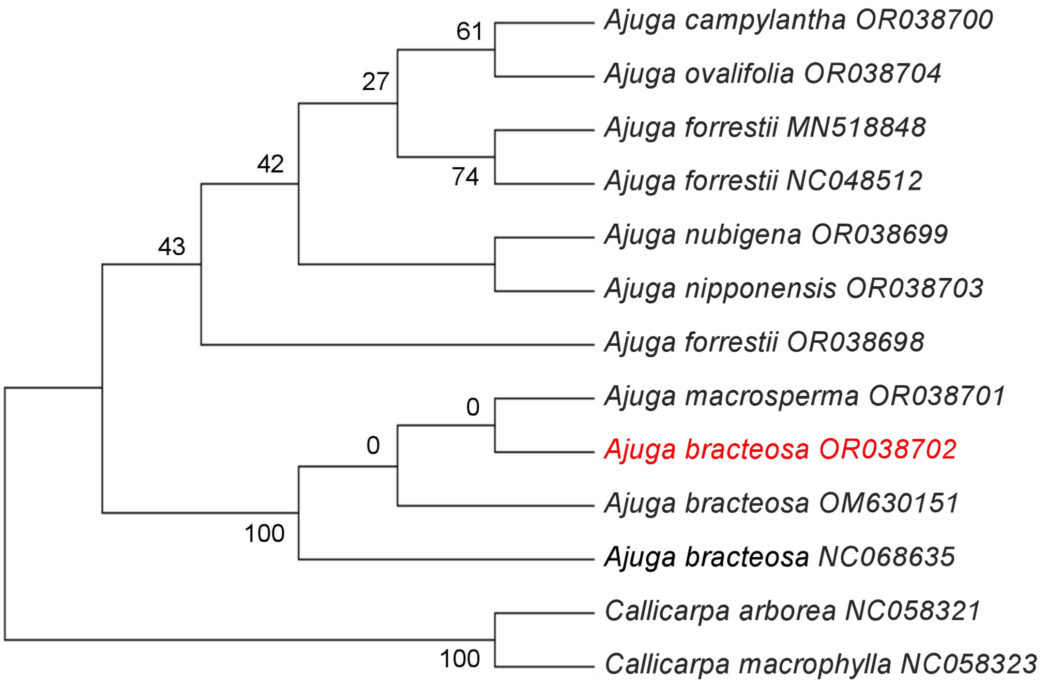

Figure S2.1 ML tree based on *accD-psaI* of cp genome.

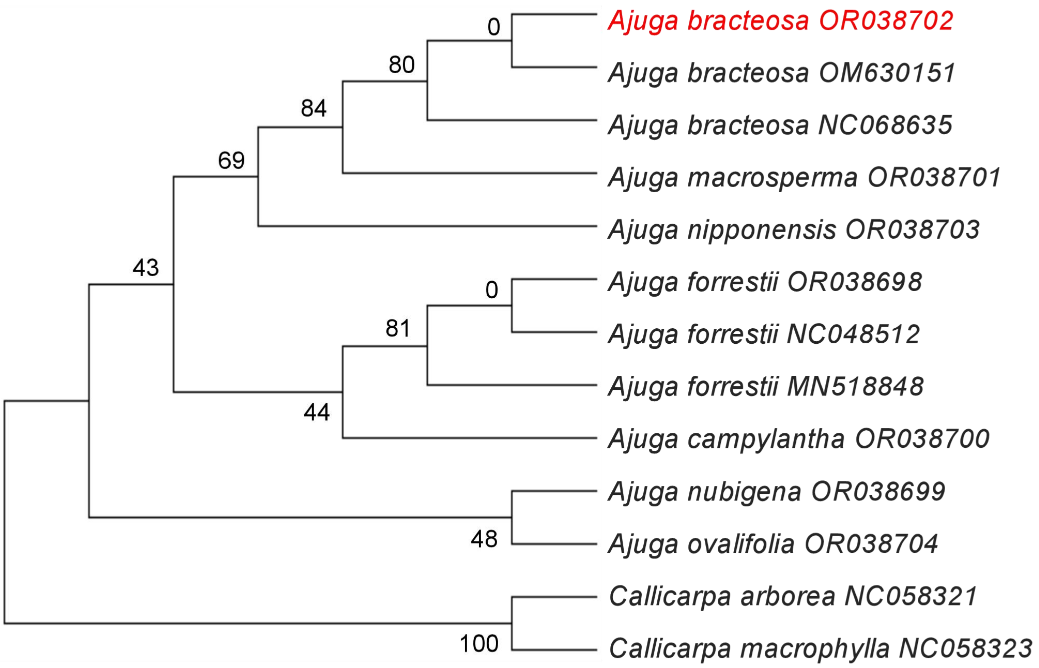

Figure S2.2 ML tree based on *atpH-atpI* of cp genome.

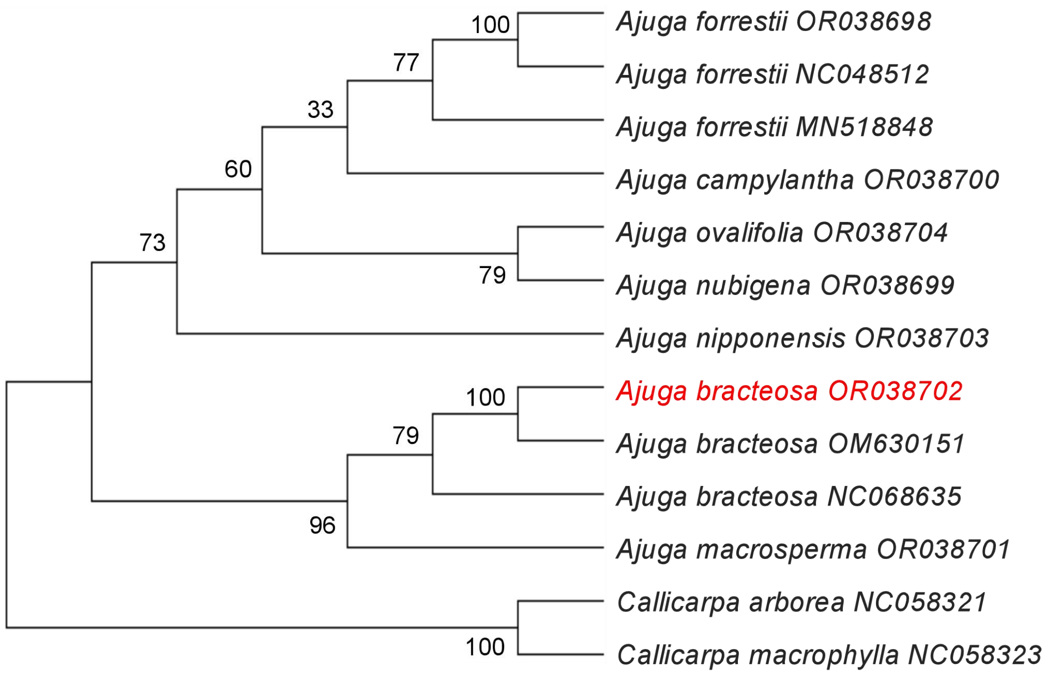

Figure S2.3 ML tree based on *ndhC-trnV*(*UAC*) of cp genome.

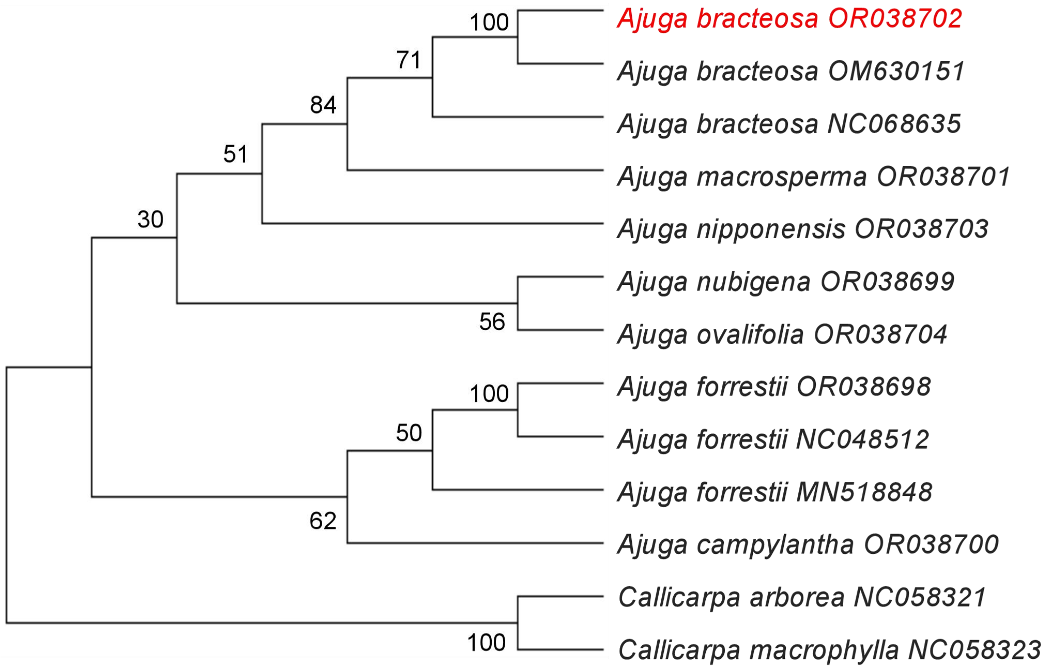

Figure S2.4 ML tree based on *ndhF-rpl32* of cp genome.

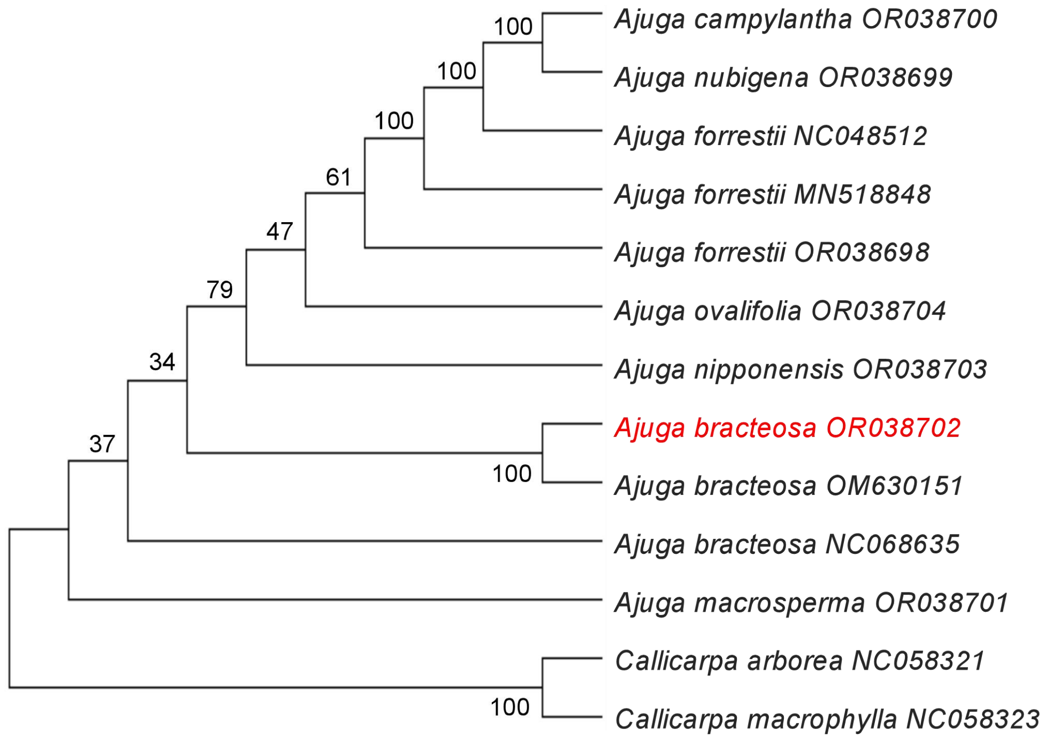

Figure S2.5 ML tree based on *rbcL-accD* of cp genome.

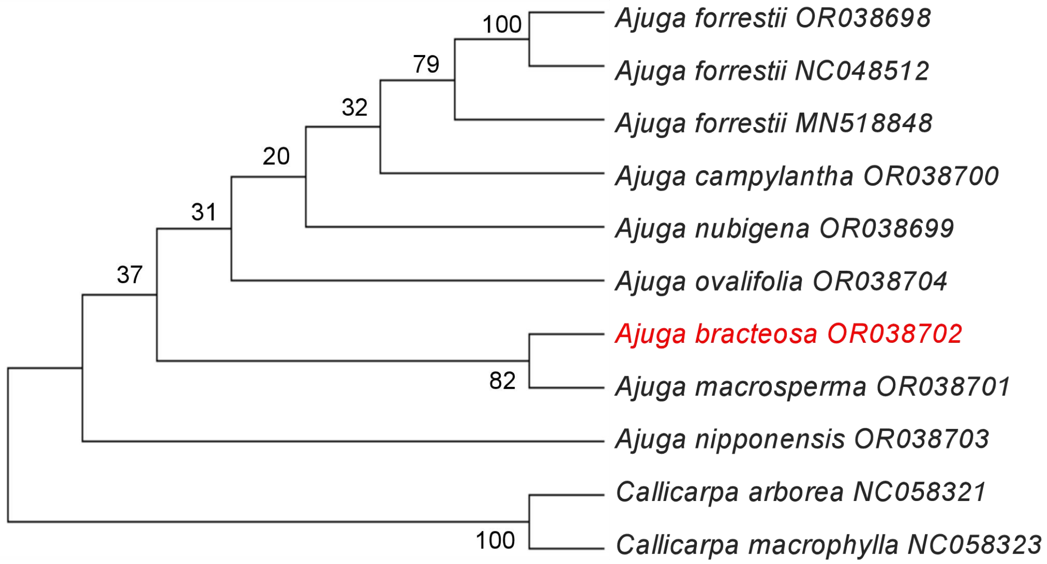

Figure S2.6 ML tree based on *rps16-trnQ*(*UUG*) of cp genome.

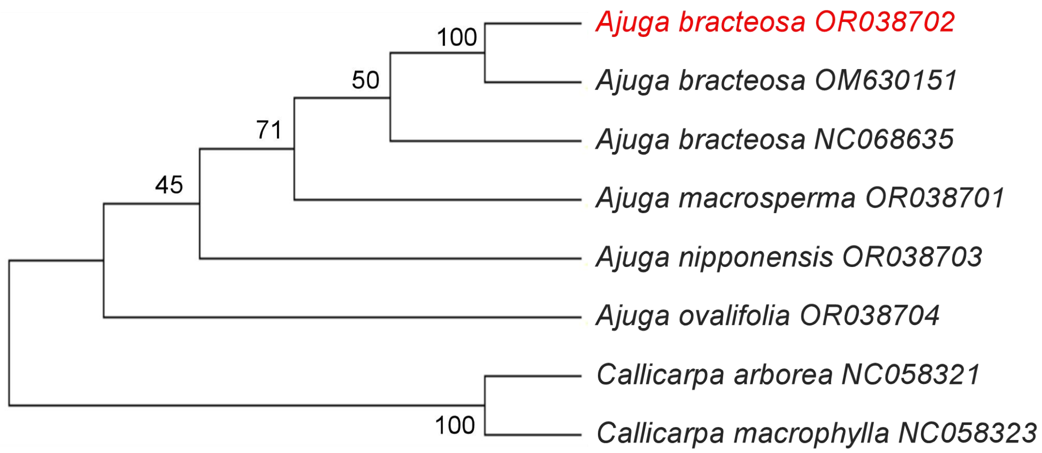

Figure S2.7 ML tree based on *trnF*(*GAA*)-*ndhJ* of cp genome.

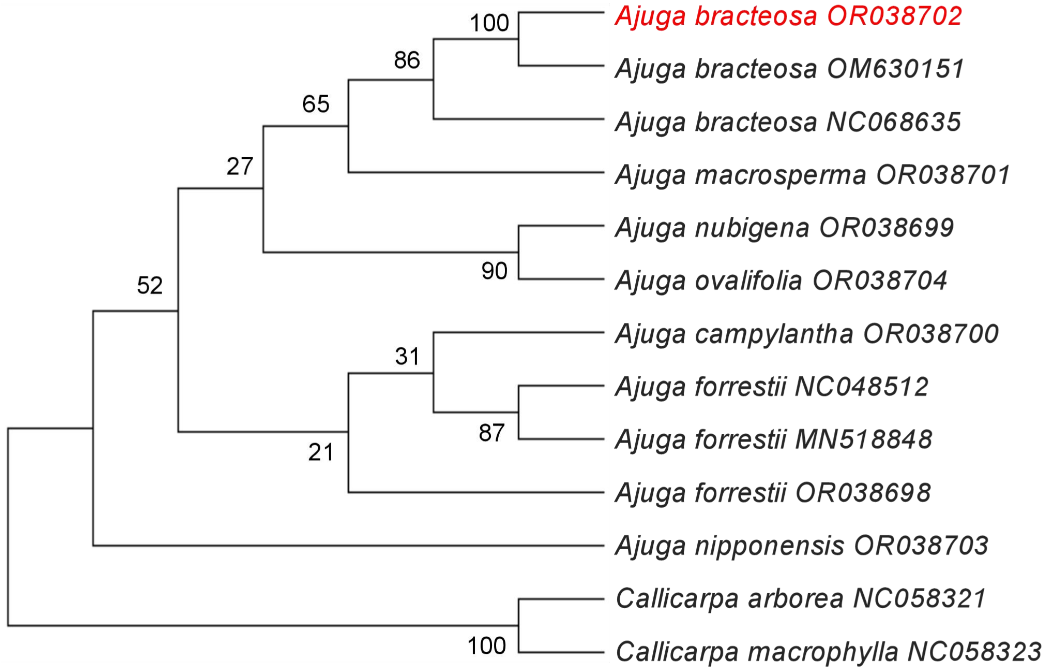

Figure S2.8 ML tree based on *trnH*(*GUG*)-*psbA* of cp genome.

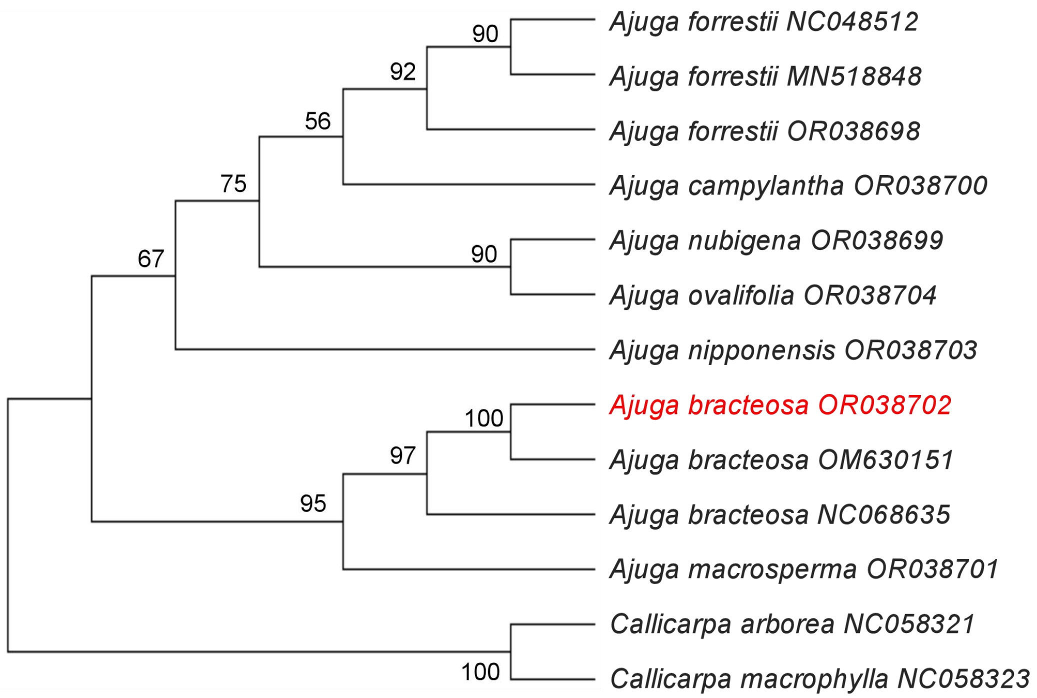

Figure S3 ML tree based on the combination of five IGSs (*accD-psaI, atpH-atpI, ndhC-trnV*(*UAC*)*, ndhF-rpl32, and trnH*(*GUG*)*-psbA*)

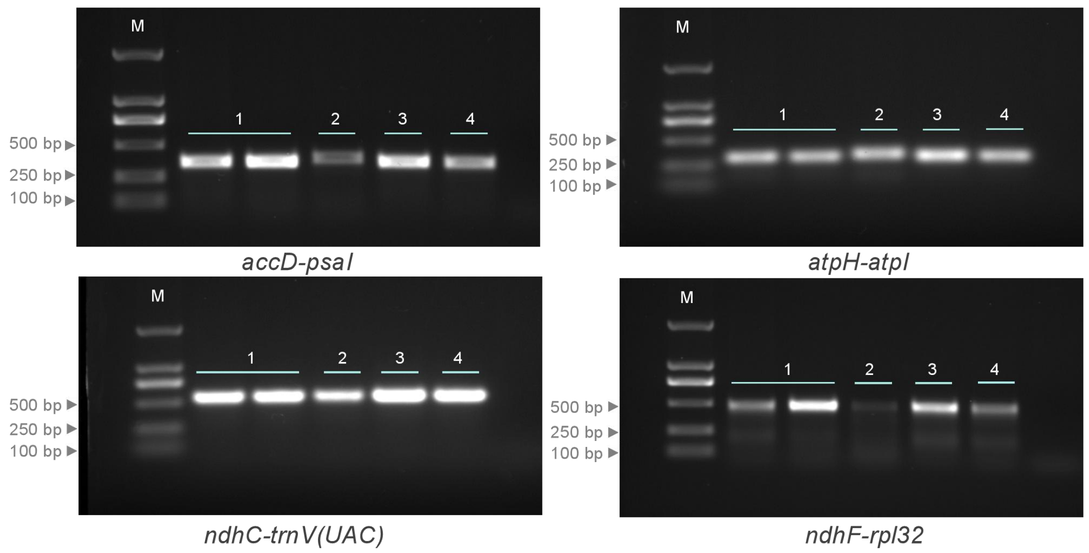

Figure S4 The gel electrophoresis results of the PCR products. Lane M was the marker of DL 2000. The lanes from left to right corresponded: 1. *A. bracteosa*; 2. *A. forrestii*; 3. *A. ovalifolia*; 4. *A. campylantha.*

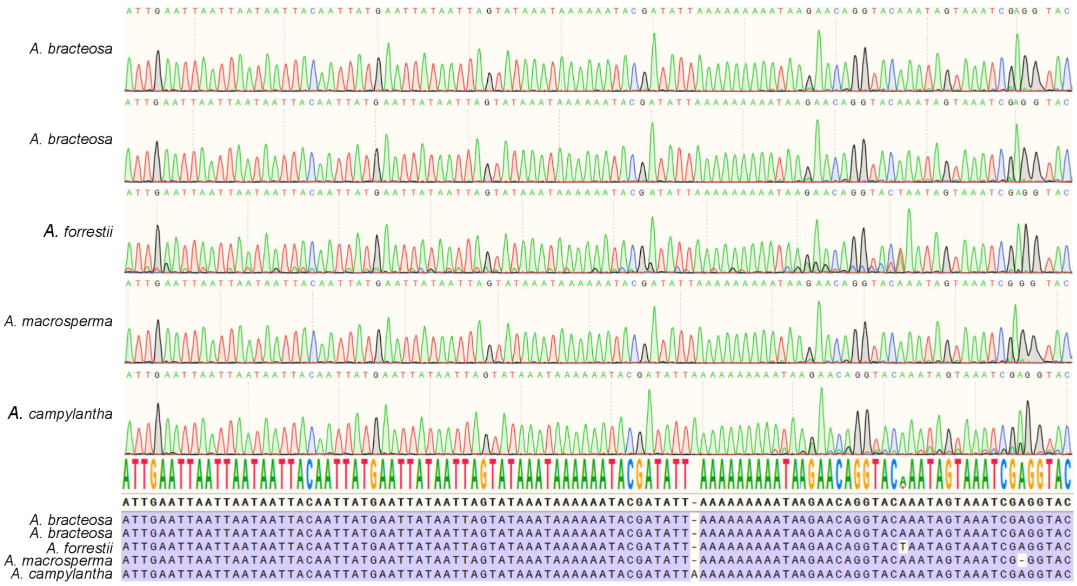

Figure S5.1 Sequencing chromatograms of the *accD-psaI* barcode in four *Ajuga* species.

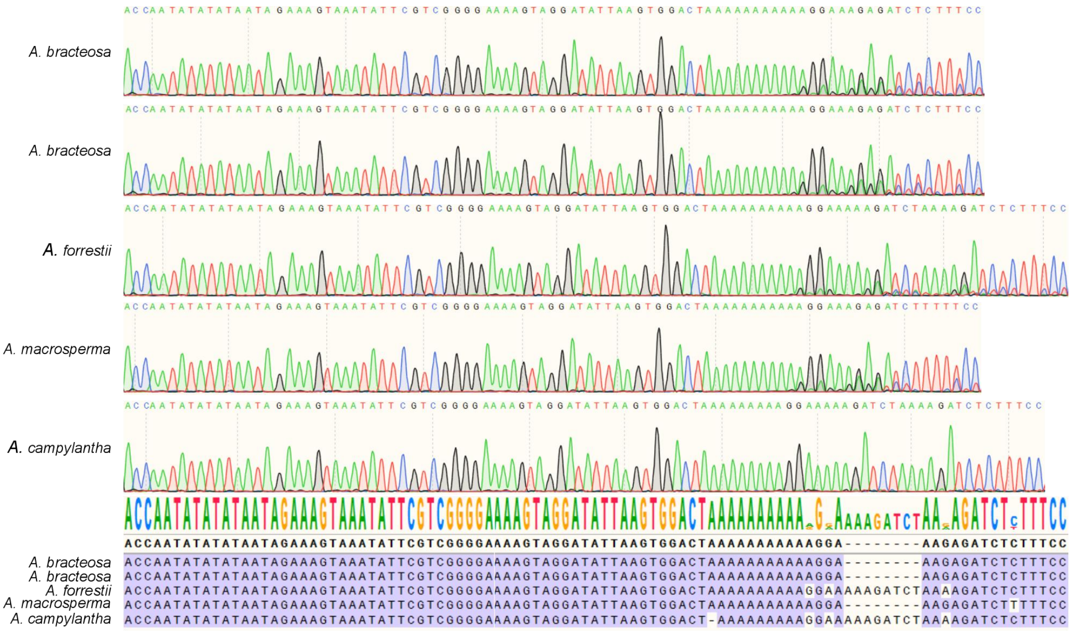

Figure S5.2 Sequencing chromatograms of the *atpH-atpI* barcode in four *Ajuga* species.

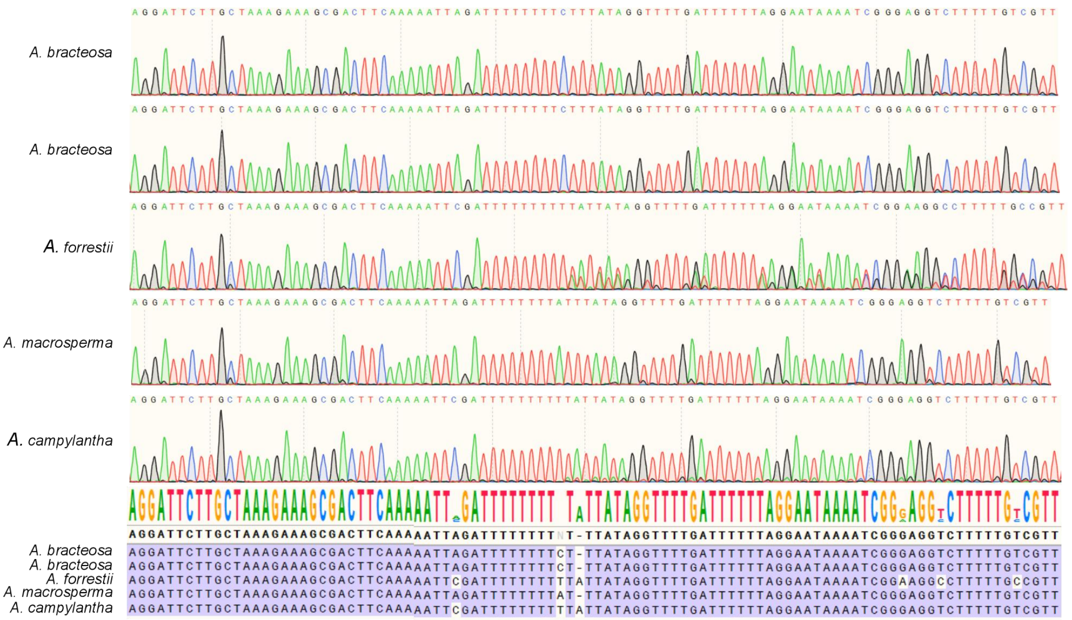

Figure S5.3 Sequencing chromatograms of the *ndhC-trnV*(UAC) barcode in four *Ajuga* species.

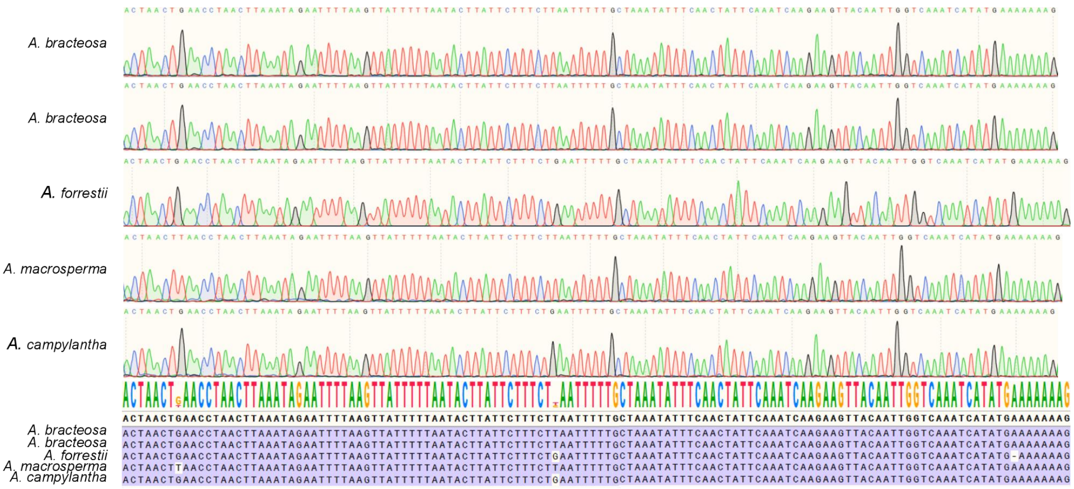

Figure S5.4 Sequencing chromatograms of the *ndhF-rpl32* barcode in four *Ajuga* species.

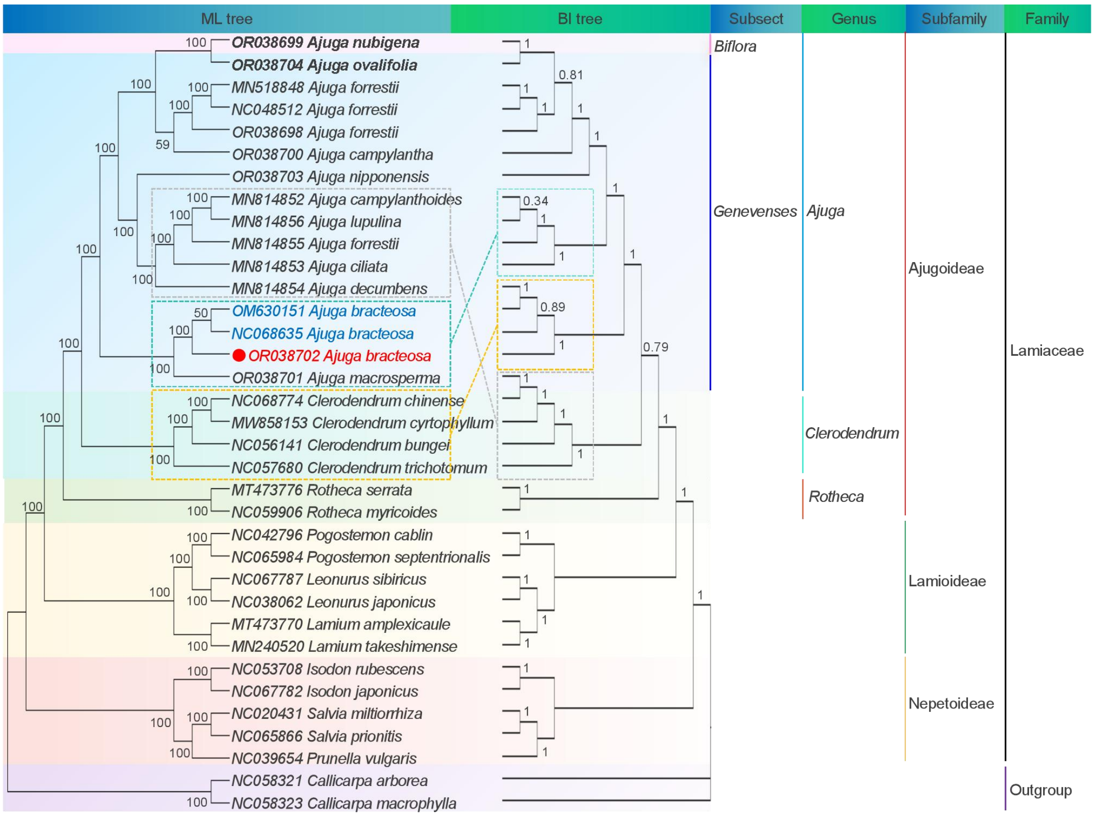

Figure S6.1 ML and BI phylogenetic tree based on cp genomes of 35 species.

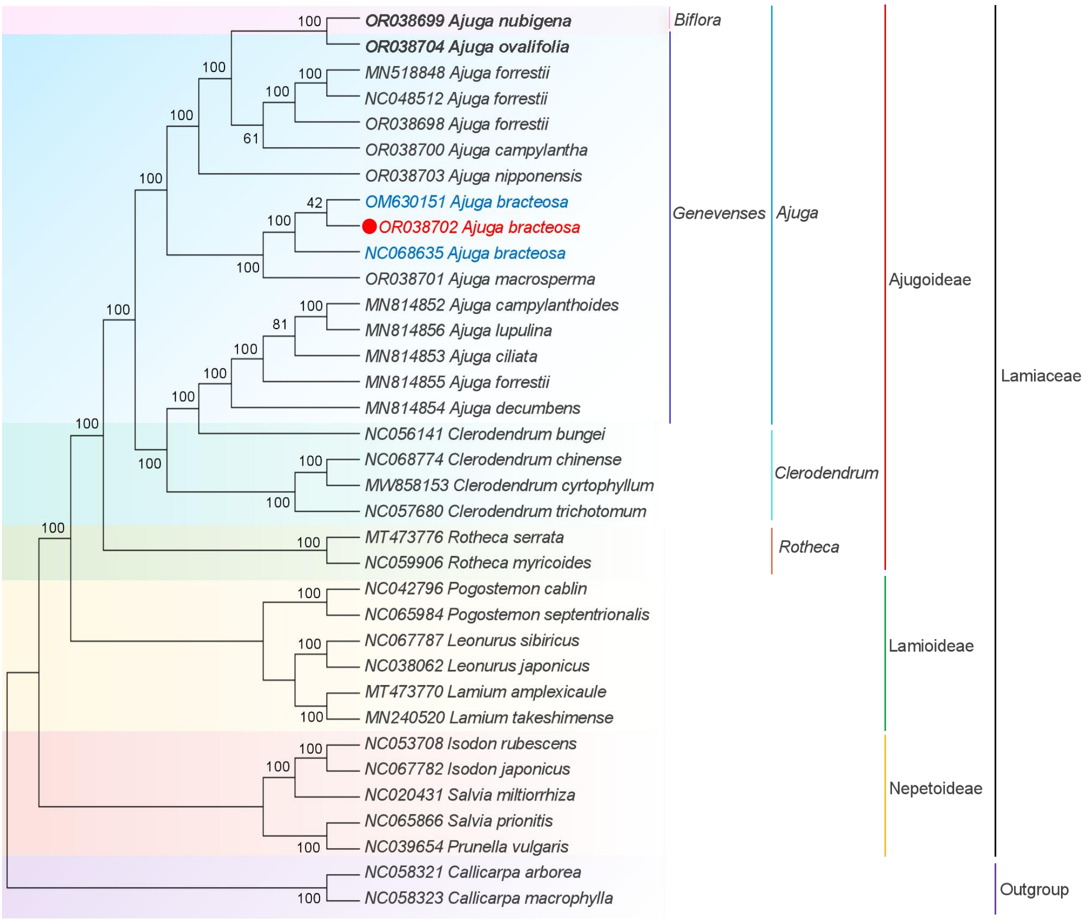

Figure S6.2 NJ phylogenetic tree based on cp genomes of 35 species.

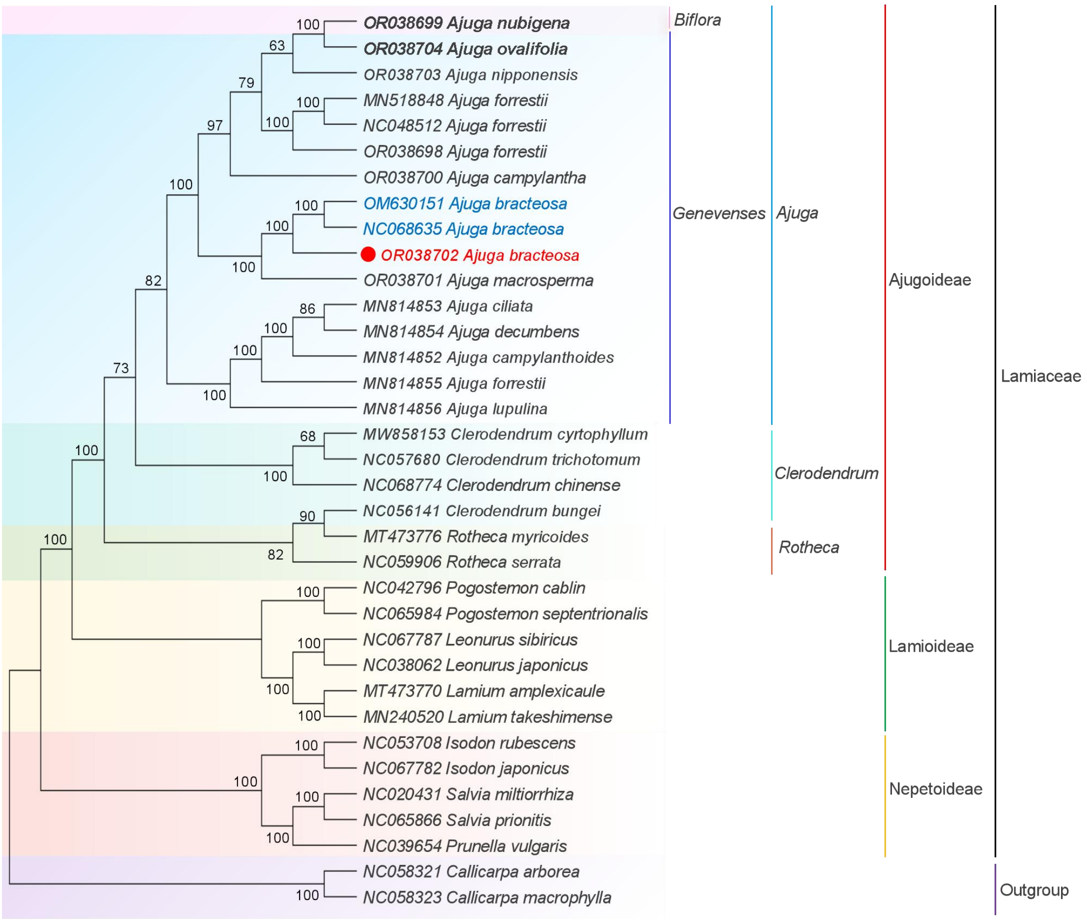

Figure S6.3 NJ phylogenetic tree based on 68 common protein-coding genes of 35 species.
